# Changes in natural killer and T lymphocyte phenotypes in response to cardiovascular risk management

**DOI:** 10.1038/s41598-023-48111-7

**Published:** 2023-11-27

**Authors:** Elin Good, Linda Åkerman, Sofia Nyström, Lena Jonasson, Jan Ernerudh, Ebo de Muinck

**Affiliations:** 1 Department of Cardiology in Linköping, and Department of Health, Medicine and Caring Sciences, Linköping University, Linköping, Sweden; 2 Department of Clinical Immunology and Transfusion Medicine, and Department of Biomedical and Clinical Sciences, Linköping University, Linköping, Sweden

**Keywords:** Cardiology, Atherosclerosis, Carotid artery disease, Risk factors, Biomarkers, Inflammation, Chronic inflammation

## Abstract

The pro-inflammatory and regulatory roles of T lymphocytes in atherosclerosis are well established but less is known about natural killer (NK) cells and natural killer T (NKT)-like cells. The effects of cardiovascular risk management on the phenotypes of these cells are unknown. To assess changes in NK cell and lymphocyte phenotypes and circulating inflammatory proteins in response to cardiovascular risk management in patients with carotid atherosclerosis. Fifty patients were included in a prospective clinical study. Measurements were at baseline and after 12 months of cardiovascular risk management. Circulating NK, NKT-like and T lymphocyte subpopulations were phenotyped by multi-colour flow cytometry. Proximity extension assay was performed for 176 plasma proteins associated with inflammation and cardiovascular disease. At 12 months there were significant reductions in LDL (P = 0.001) and blood pressure (P = 0.028). NK cells responded with a reduction in pro-inflammatory (NKG2C^+^) cells (P = 0.0003), an increase in anti-inflammatory (NKG2A^+^) cells (P = 0.032), and a reduction in terminally differentiated (CD57^+^) NK cells. NKT-like cells showed a similar decrease in terminally differentiated subpopulations (P = 0.000002). Subpopulations of T helper cells exhibited a significant reduction in central memory (P = 1.09 × 10^−8^) and a significant increase in CD4^+^ naïve- (P = 0.0008) and effector memory T cells (P = 0.006). The protein analysis indicated that cardiovascular risk management affects proteins involved in the inflammatory NF-κB pathway. The consistent decrease in senescent phenotypes of NK, NKT-like and CD4^+^ cells with a concomitant increase in more naïve, phenotypes suggests a change towards a less pro-inflammatory lymphocyte profile in response to cardiovascular risk management.

**Trial registry name**: CARotid MRI of Atherosclerosis (CARMA). ClinicalTrials.gov identifier NCT04835571 (08/04/2021). https://www.clinicaltrials.gov/study/NCT04835571.

## Introduction

Cardiovascular risk management combines pharmacological therapy with lifestyle optimization to prevent cardiovascular disease^1^. It is the cornerstone of cardiovascular disease prevention. However, many of the mechanisms that might explain its beneficial effect, remain to be elucidated. Natural killer (NK) cells (CD3^−^CD56^+^) and Natural Killer T-like (NKT-like) cells (CD3^+^CD56^+^) have emerged alongside T lymphocytes as important contributors to the inflammatory response in atherosclerosis^2–4^. In atherosclerosis all three cell lineages are polarized towards a senescent, pro-inflammatory cell phenotype^4–7^. We hypothesized that cardiovascular risk management would shift circulating NK cells, NKT-like cells, and T cell subpopulations towards a more naïve, ‘rejuvenated’ cell phenotype. The current study focuses on patients with carotid atherosclerosis because these patients are at very high risk for future cardiovascular events and we have previously shown that cardiovascular risk management for these patients is deficient in all aspects^8^. Thus, we designed the current study to address the unmet clinical need for improved cardiovascular risk management in patients with carotid atherosclerosis while at the same time asking mechanistic questions about the effect of this approach on lymphocyte phenotypes.

In the thymus, most T cells develop into CD4^+^ T helper (Th) or CD8^+^ T cytotoxic (Tc) cells. These T cell populations are the foundation of the adaptive immune response. NK cells, on the other hand, are derived directly from the bone marrow and are part of the innate immune system. NKT-like cells are hybrid cells expressing surface markers of both T- and NK-cells^9^. They can be regarded as highly differentiated T cells co-expressing CD56 along with other NK receptors.

Studies in mouse models of atherosclerosis have shown that NK cells accelerate atherosclerosis through the secretion of the cytotoxic proteins perforin and granzyme, and the release of IFN-γ^3^. The transition to an activated pro-atherosclerotic NK cell phenotype has been shown to be driven by Interleukins (IL-12, IL-15, IL18)^10,11^. Evidence for a role of NK cells in human atherosclerosis comes from plaques of patients with carotid atherosclerosis. NK cells that were isolated from plaques of symptomatic patients were stronger producers of IFN-γ than those isolated from patients with asymptomatic plaques^12^.

Mechanistic evidence for a role of NKT-like cells in atherosclerosis also comes from mouse models. NKT-like cells are activated when exposed to lipids presented by antigen presenting cells such as dendritic cells and macrophages. The lipids are bound to the CD1d-β_2_ macroglobulin complex on these cells. The activated NKT-like cells secrete cytokines associated with T helper 1 (Th1), T helper 2 (Th2) and T helper 17 (Th17) responses^4^. Furthermore, IL-12 and IL-18 from antigen presenting cells induce NKT-like cells to produce IFN-γ^13^. In human atherosclerosis, plaques show co-localization of NKT-like cells and dendritic cells^14^. Also, IFN-γ producing NKT-like cells were found to be persistently accumulated in blood from patients with coronary artery disease^6^.

Several studies have shown beneficial effects of statin treatment on T lymphocyte profiles, demonstrating an increase in anti-inflammatory T helper cells and Th2 cells^15–17^. However, there are no comprehensive studies that evaluate how sub-lineages of CD4^+^ and CD8^+^ T lymphocytes respond to statin treatment. Importantly, statin therapy alone does not fulfil the treatment goals for patients with advanced atherosclerosis. According to European guidelines, patients with very high cardiovascular risk should be offered personalised cardiovascular risk management. This approach combines pharmacological treatment of hyperlipidaemia, hypertension and diabetes mellitus with life style optimization that includes smoking cessation, dietary changes and increased physical activity^18^. Therefore, the effect of comprehensive cardiovascular risk management was evaluated instead of statin therapy only.

Here, in patients with carotid atherosclerosis, we assessed the effect of cardiovascular risk management on subpopulations of NK cells, NKT-like cells, and T lymphocytes. In addition, circulating proteins were measured to explore changes in protein-mediators of inflammation and cardiovascular disease.

## Methods

### Ethical approval

This study was registered at ClinicalTrials.gov under identifier NCT04835571 (08/04/2021). The study protocol was approved by the Swedish Ethical Review Authority (approval nr.: 2016-441-31, nr. 2019-03969 and nr. 2021-04334). The study was performed in accordance with the Declaration of Helsinki. All participants gave their written informed consent before participation.

### Patients

Study participants were selected from an ultrasound database for all carotid duplex ultrasound investigations that were performed in the region between 2012 and 2016. Selection was based on duplex ultrasound criteria for stenosis severity based on the maximum flow velocity at the point of maximal luminal narrowing. A cut-off was chosen at doppler flow velocity ≥ 1.3 m/s, which corresponds to a > 50% stenosis^1,19^. Patients aged 18–80 years were included. Exclusion criteria were inability to give informed consent, inability to participate in examinations necessary for the study, stroke ≤ 1 month of inclusion, co-diseases or factors that inhibited optimization of risk factor management, immunologic disorders, neoplastic diseases, and patients treated with immunosuppressive/anti-inflammatory agents. Data were retrieved from the electronic patient record, and data were also collected through clinical examination, patient interviews, and blood sample analysis.

### Clinical assessment and cardiovascular risk management

Participants were invited to a study visit at baseline and at follow-up after 12 months. A study visit included clinical cardiovascular assessment and a patient interview by the study clinicians (E.G and E.d.M). Blood samples were taken, and blood pressure was measured by a research nurse. Blood samples were obtained in EDTA tubes. One blood sample was used for routine laboratory tests, a second sample for plasma isolation and a third sample was used for immediate lymphocyte immunophenotyping.

At the first study visit a thorough assessment of the patient’s cardiovascular risk factors and lifestyle was performed. The purpose was to identify all modifiable risk factors, so that the clinicians could provide a personalised plan for each patient that would address comprehensive risk factor management and lifestyle optimization according to the cardiovascular prevention guidelines current at the time^18^. The treatment targets for patients with a high cardiovascular risk, such as the current cohort, were as follows: blood pressure ≤ 140/90 mmHg, antiplatelet therapy (or anticoagulants for those with co-morbidities requiring such treatment), LDL ≤ 1.8 mmol/L, adequate physical exercise (exercise of medium intensity for at least 30 min per day, 5 days a week, or exercise of very high intensity for at least 15 min per day, 5 days a week), healthy diet (low intake of saturated fats, high intake of whole grain products, vegetables, fruits and fish), weight control (preferably BMI 20–25 kg/m^2^), smoking cessation, low alcohol consumption. LDL targets for high-risk patients in international guidelines have been sharpened during the period between data collection and publication, but the study aimed for the targets that were recommended at the time of inclusion.

Because of the known influence of cytomegalovirus (CMV) infection on CD8^+^ T cell status, IgG- and IgM anti-CMV antibody titres were measured at baseline and at follow-up.

### Questionnaires

A self-report questionnaire, comprising 35 questions, was used to collect detailed information on health, family history and medication. This was a truncated version of the general self-report form used in the Swedish CardioPulmonary bioImage Study^20^. In the follow-up questionnaire 14 questions were added to assess lifestyle changes over the study year and to detect impediments for the implementation of cardiovascular risk management.

### Monoclonal antibodies and flow cytometry

Lymphocytes were stained with fluorochrome-conjugated antibodies against human immune cells. A list of the antibodies used in the study is presented in Supplementary Table S1A.

Using flow cytometry, absolute numbers (cells/μL) of lymphocytes, T-, B, and NK cells in EDTA whole blood were determined by using Becton, Dickinson, and Company (BD) Multitest reagent with BD Trucount tubes and BD FACS Lysing solution, all from BD Biosciences (San Jose, CA, USA), according to manufacturer’s instructions. For staining of T- and NK cell markers, 50 µL of EDTA whole blood was incubated for 15 min with monoclonal antibodies (for info on each panel, see Supplementary Table S1B). Erythrocytes were lysed for 15 min using BD FACS lysing solution and the cells were washed in 2 ml PBS with 2% albunorm, Human albumin solution (Octapharma, Lachen, Switzerland), resuspended in 500 µL PBS/HSA and immediately acquired on a FACSCanto II using FACSDiva (BD Biosciences). For staining of regulatory T cells, 400 µL of whole blood was first incubated for 15 min at room temperature with monoclonal antibodies for extracellular staining. Cells were then fixed and permeabilized using eBioscience Intracellular Fixation & Permeabilization Buffer Set (Invitrogen, CA, USA). Fixed and permeabilized cells were intracellularly stained with PE anti-Foxp3 and APC anti-CTLA-4, washed, reconstituted in PBS/HSA, and immediately acquired on a FACSCanto II.

### Gating strategy of lymphocytes and lymphocyte subpopulations

Analysis of lymphocytes for absolute cell counts was performed in FACSDiva, while Kaluza Analysis Software version 2.1 (Beckman Coulter, Miami, USA) was used for analysis of lymphocyte subsets. Natural killer cells (CD3^-^CD56^+^) were divided into mature (CD56^dim^) and immature (CD56^bright^), and the mature population was assessed for expression of the differentiation marker CD57, the inhibitory receptors NKG2A and CD85j and the activating receptor NKG2C (Supplementary Fig. S1B). The population of NKT-like cells was identified by the simultaneous expression of CD3 and CD56, as well as CD3^+^CD8^+^ NKT-like cells, which were both assessed for expression of CD57 (Supplementary Fig. S2). Lymphocytes were identified either by CD45 and side scatter (SSC) or by forward scatter (FSC) and SSC. Differentiation status of T helper and cytotoxic T cells was determined by surface expression of CD45RA and CD62L, whereas activation status was assessed by expression of HLA-DR (Supplementary Fig. S3).

Two patients were excluded from analysis of naïve T cells, central memory cells (T_CM_), effector memory cells (T_EM_) and terminally differentiated effector memory cells (T_EMRA_), due to a variant pattern of CD45RA expression, which is known from the literature to be present in up to 8% of individuals^21^. In addition, data from nine patients were excluded because of a technical error of the CD45RA antibody. The technical error also excluded the possibility to identify Tregs and their subpopulations with sufficient precision based on their expression of CD45 and FoxP3 as suggested by Miyara et al.^22^. For this reason, Tregs were instead defined as CD4^+^CD25^+^FoxP3^+^ (Supplementary Fig. S4)^2,4^.

### Protein profiling by proximity extension assay

EDTA plasma was obtained by centrifugation, which was performed within two hours after sampling. Aliquots of plasma were stored at − 80° until use. Plasma samples were thawed and pipetted to microtiter plates that were re-frozen and shipped to the Clinical Biomarker Facility at the SciLifeLab in Uppsala, Sweden. The proximity extension assay (PEA) technology (Olink AB, Uppsala, Sweden) was used for protein profiling of samples from baseline and follow-up. The Cardiovascular Panel (Olink Target 96 Cardiovascular II, v.5007) with 92 pre-selected proteins (Supplementary Table S2) and the Inflammation Panel (Olink Target 96 Inflammation, v.3023) with 92 pre-selected proteins (Supplementary Table S3) were used. The PEA technology is based on dual recognition of protein epitopes using pairs of antibodies equipped with DNA single-strand oligonucleotide reporter molecules. If the pair of antibodies bind their respective target in close proximity on the same protein, this leads to a hybridization of the oligonucleotides and the generation of a double-stranded DNA amplicon, which is quantified using quantitative polymerase chain reaction (qPCR). The assay includes internal controls, and inter-plate variability was adjusted by intensity normalization. Relative plasma protein abundance is expressed as normalized protein expression (NPX) in log2-format. The log-2 scale implicates that an increase in one NPX unit corresponds to a doubling of the protein content.

### Statistical analysis

SPSS Statistics 28 (International Business Machines Corporation, New York, NY, USA) was used for statistical analysis. Continuous parameters were presented as means ± standard deviation (SD) and for categorical variables frequencies with percentages were used. Differences between groups were calculated by independent samples Student’s t-test for continuous variables. Differences between groups regarding binary data were calculated using the chi-square test. Differences over the study year for patients were calculated using paired samples t-test. Pearson correlations and simple linear regression were used to explore the relationship between treatment outcomes, routine lab, immune cells, and cytokines. A subgroup analysis was performed based on CMV status, to assess differences in cellular responses in CMV positive versus CMV negative subjects. P-values < 0.05 were considered statistically significant. A false discovery rate (FDR) test according to Benjamini–Hochberg was used for the protein enrichment analysis.

### Functional annotation and protein interaction analyses

Over-representation pathway enrichment analysis of differently expressed plasma proteins was performed with the WebGestalt online tool^23^. Protein–protein interaction networks were created with differentially expressed proteins using STRING version 11.5^24^ by including interactions with confidence score > 0.250. STRING database is a curated knowledge database of known and predicted protein–protein interactions^25^.

## Results

### Patients had a high cardiovascular risk burden

In total, 52 patients participated in the study, but two withdrew prior to completion of the study. Therefore, results from 50 patients were analysed. See Supplementary Fig. S5 for inclusion flow chart and Supplementary Fig. S6 for an overview over excluded patients. Reasons for discontinuation were lack of time and unwillingness to participate in further examination. Lipid levels (total cholesterol, HDL, LDL, NONHDL, Apolipoprotein A1, Apolipoprotein B1) and baseline characteristics (age, weight, length, BMI, waist circumference, comorbidities) were previously published in a study assessing the relationship between circulating lipids and plaque composition as measured by MRI^26^.

Patients had a mean age of 72 (± 5) years, 34% were female (Table 1). Lifestyles and diseases associated with high cardiovascular risk were common in this cohort. The most common risk factors were hypertension (84%), body mass index > 25 (84%), and LDL > 1.8 mmol/L (66%) (Supplementary Table S4). Baseline characteristics were similar for the 33 male and 17 female patients in the study, with no significant differences in risk factors including lipid levels (total cholesterol P = 0.424, LDL P = 0.518). Kidney function deteriorated significantly during the study year, with an increase in creatinine from 86 to 90 mmol/L (P = 0.011) and an increase in urea (Supplementary Table S8).

**Table 1:** Baseline characteristics.

Patient characteristics	
Age (mean, SD)	72.4 (5.1)
Male sex (N, %)	33 (66.0)
Body Mass Index (mean, SD)	28 (4.3)
Waist, cm (mean, SD)	100 (12.7)
Comorbidities (N, %)	
Cerebrovascular disease	31 (62.0)
Heart failure	2 (4.0)
Ischemic heart disease	13 (26.0)
Atrial fibrillation	7 (14.0)
Hypertension	42 (84.0)
Peripheral artery disease	13 (26.0)
Aortic disease	1 (2.0)
Diabetes mellitus	12 (24.0)
Chronic pulmonary disease	5 (10.0)

Baseline characteristics for the 50 patients included in the study.

Regarding CMV status, none of the patients had titres compatible with a reactive CMV IgM response. For all patients IgG status remained unchanged from baseline to follow-up and IgG antibodies were detected at both time points in 39 patients (78%). As expected, higher counts of cytotoxic T cells were detected in CMV positive patients compared to those who were CMV negative (P = 0.006), but these numbers showed no significant change over the study year. For T helper cells there were no significant differences between the groups, except that those who were CMV-positive had higher levels of CD28^-^ cells both at baseline (P = 0.017) and at follow up (P = 0.001). The proportion of differentiated NKT-like cells (CD3^+^CD56^+^CD57^+^) was higher in CMV positives compared to CMV negative patients, both at baseline and after 12 months. Collectively, an expected effect of CMV status was noted, and this skewing remained unchanged over the study period. All CMV subgroup results are presented in Supplementary Tables S5–S7.

### Cardiovascular risk management

A majority of patients had some degree of pharmacological cardiovascular prevention prior to the start of the study, including statins, anti-hypertensives and platelet inhibitors, but the intervention resulted in an improved risk profile at group level with significantly lowered systolic- and diastolic blood pressure (Table 2) and significantly reduced LDL (Table 3, and for complementary lab results Supplementary Table S8). This was achieved through adjustments in pharmacological treatment, resulting in changes in medications for 72% of the patients. However, the treatment targets were not achieved for all patients (Table 2). Only 13 individuals (27%) reached targets for blood pressure, lipids, non-smoking, and physical activity combined. Regression analysis did not show any strong linear correlations that could be interpreted as functionally relevant between treatment outcomes, routine lab, immune cell populations and cytokines, as shown in Supplementary Table S9. This was also true for the acute-phase reactant hsCRP. There was a modest non-significant reduction in hsCRP (Table 3) and, the only statistically significant correlations were in relation to CD3^+^ (P = 0.049, Pearson correlation 0.279) and CD4^+^8^+^ (P = 0.01, Pearson correlation 0.462). Changes in medical treatment during the study year are presented in Supplementary Table S10.

**Table 2:** Changes in clinical parameters, risk factors and lifestyle measures over the study year.

	Baseline	Follow-up	Difference	P
Clinical data (N, %)				
Weight (kg)	81.2 (± 15.1)	80.7 (± 15.0)	− 0.5 (± 2.7)	0.248
Waist (cm)	100 (± 12.7)	100 (± 12.2)	− 0.1 (± 3.0)	0.887
BMI	28.4 (± 4.3)	28.2 (± 4.6)	+ 0.2 (± 1.8)	0.456
Systolic blood pressure^a^ (mmHg)	150 (± 18)	143 (± 19)	− 6.3 (± 19.7)	0.028
Diastolic blood pressure^a^ (mmHg)	84.2 (± 9.2)	81.1 (± 9.4)	− 3.1 (± 7.7)	0.006
Systolic home pressure (mmHg)	135 (± 13)	130 (± 13)	− 4.8 (± 9.9)	0.010
Diastolic home pressure (mmHg)	76 (± 6)	74 (± 7)	− 2.2 (± 5.1)	0.019
Frailty score	3.5 (± 0.6)	3.4 (± 1.0)	− 0.1 (± 1.0)	0.673
Risk factors (N, %)				
Active smoking	7 (14)	6 (12)	− 1 (2)	0.794
Systolic blood pressure > 140 mmHg	36 (62)	29 (58)	− 7 (14)	0.499
LDL > 1.8	33 (66)	24 (48)	− 9 (18)	0.341
HbA1c > 53 mmol/mol	7 (14)	4 (8)	− 3 (6)	0.390
Levels of physical exercise				
No physical activity	10 (20)	14 (28)	+ 4 (8)	0.463
Limited physical activity	27 (54)	23 (46)	− 4 (8)	0.644
Regular physical exercise	10 (20)	11 (22)	+ 1 (2)	0.843
Consistent physical exercise	3 (6)	2 (4)	− 1 (2)	0.663

Changes in risk factors and lifestyle measures for the 50 patients in the study. Data for levels of physical exercise were based on a questionnaire, filled in by the clinician during the patient interview at baseline and follow-up visits.

^a^Right arm, sitting position.

**Table 3:** Blood test results.

Test (mmol/L)	Baseline	Follow-up		
	Mean (SD)	Mean (SD)	Mean difference (SD)	*p*
Hb	142 (± 11.1)	141 (± 10.9)	− 1.14 (± 7.20)	0.268
Leukocytes	6.49 (± 1.36)	7.12 (± 1.66)	+ 0.63 (± 1.16)	0.00034
Thrombocytes	220 (± 50.2)	218 (± 44.2)	− 2.42 (± 26.9)	0.528
Eosinophils	0.26 (± 0.18)	0.29 (± 0.30)	+ 0.03 (± 0.25)	0.359
Basophils	0.05 (± 0.03)	0.07 (± 0.03)	+ 0.01 (± 0.03)	0.008
Monocytes	0.61 (± 0.19)	0.66 (± 0.18)	+ 0.06 (± 0.14)	0.008
Neutrophils	3.83 (± 1.02)	4.43 (± 1.42)	+ 0.61 (± 1.01)	0.0001
Lymphocytes	2.03 (± 0.47)	2.05 (± 0.51)	+ 0.02 (± 0.34)	0.680
Neutrophil:lymphocyte ratio	1.95 (± 0.61)	2.29 (± 0.92)	+ 0.34 (± 0.71)	0.001
Cholesterol, total	4.14 (± 1.13)	3.79 (± 1.05)	− 0.35 (± 0.72)	0.001
Triglycerides	1.25 (± 0.50)	1.21 (± 0.57)	− 0.038 (± 0.36)	0.455
HDL	1.41 (± 0.46)	1.35 (± 0.46)	− 0.07 (± 0.23)	0.050
LDL	2.16 (± 0.96)	1.88 (± 0.83)	− 0.28 (± 0.56)	0.001
NONHDL	2.71 (± 1.09)	2.43 (± 1.00)	− 0.28 (± 0.63)	0.003
ApolipoproteinA1	1.49 (± 0.31)	1.48 (± 0.32)	− 0.01 (± 0.19)	0.782
ApolipoproteinB1	0.84 (± 0.26)	0.78 (± 0.27)	− 0.06 (± 0.16)	0.008
ApoB/ApoA	0.61 (± 0.25)	0.54 (± 0.21)	− 0.07 (± 0.16)	0.004
hsCRP (median [iq range])	1.5 (2.5)	1.3 (2.8)	− 0.15 (1.02)	0.447
HbA1c	43.2 (± 13.0)	41.1 (± 10.5)	− 2.11 (± 10.8)	0.174
f-Glucose	6.22 (± 1.45)	6.70 (± 4.19)	+ 0.48 (± 4.29)	0.498

Blood test results at baseline and at follow-up. Paired samples T test was used for statistics, to test for significant differences over the study year.

Lifestyle counselling aimed to encourage smoking cessation, increase physical activity, and improve diet, but there was no significant change regarding these parameters. We encouraged weight reduction, but no significant weight reduction was observed (P = 0.248). In ten patients there were neither medical adjustments, nor improvements in dietary- and exercise lifestyle habits. Changes in clinical data and levels of physical exercise are shown in Table 2.

Study physicians remained in continuous contact with the patients throughout the study year and surveyed blood tests and blood pressure measurements while adjusting patient management accordingly. Despite these efforts, not all patients achieved all prevention targets. Patient adherence to the medical and lifestyle regimen was assessed through detailed questionnaires, the results of which are summarized in Supplemental Table S11. Four patients (8%) were unwilling to adjust their medication, five (10%) expressed no motivation to change their exercise habits and six (12%) refused to stop smoking. Furthermore, there were medication side effects that precluded optimal pharmacological management and there were impediments to implement lifestyle changes, with bodily pain being the most common impediment to regular exercise (Supplementary Table S11).

### Immune phenotyping of lymphocytes and lymphocyte subpopulations

**Figure 1:**
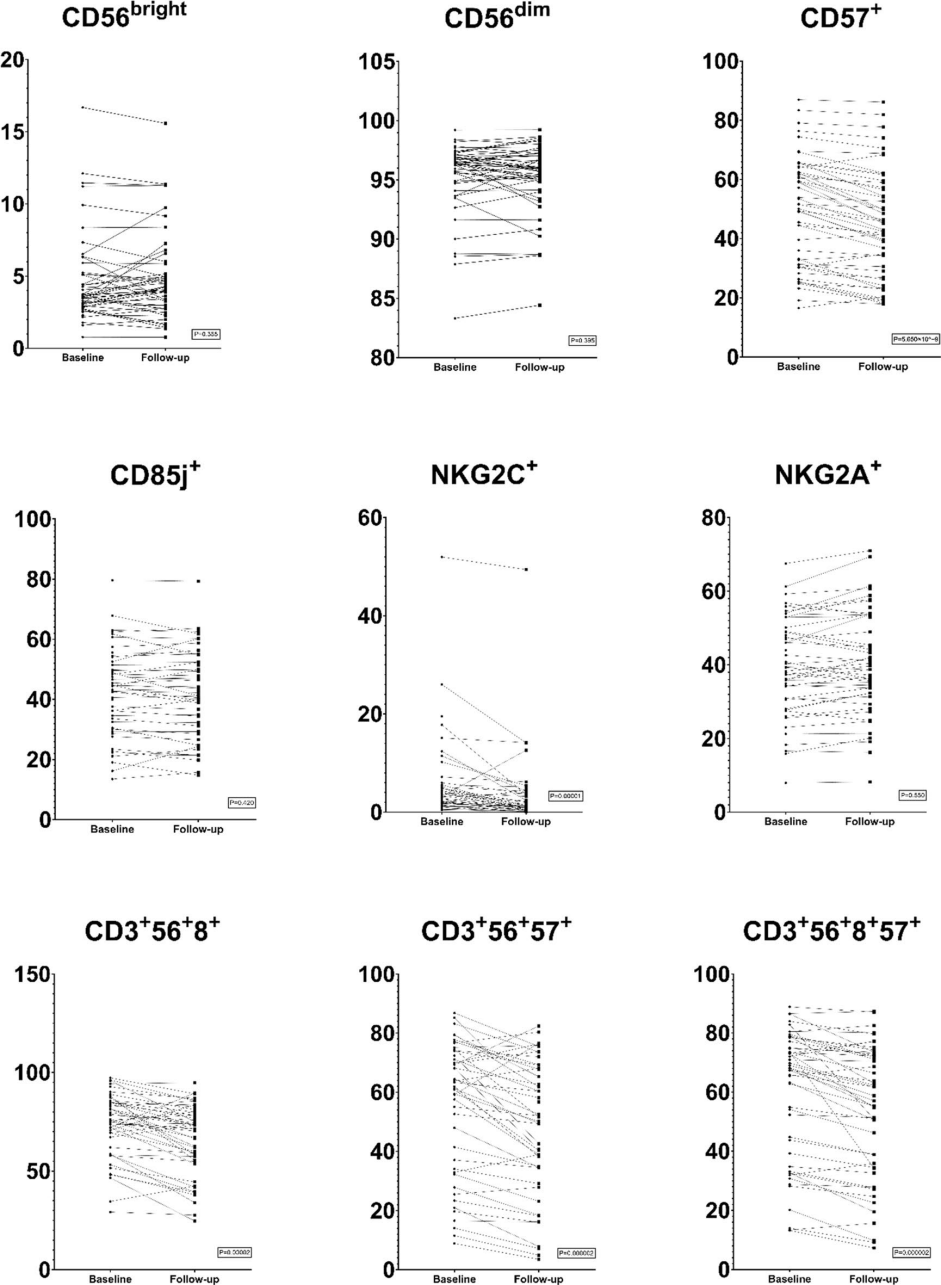
Natural Killer (NK) cell subsets and Natural Killer T-like (NKT-like) cell subsets in patients before and after cardiovascular risk management. Responses are presented for all 50 patients, visualizing changes between baseline and follow-up for each individual. The two upper rows show NK-cell subset response. CD56^bright^ and CD56^dim^ are expressed as % of NK cells. CD57^+^, CD85j, NKG2C^+^ and NKG2A^+^ and are expressed as % of mature (CD56dim) NK cells. The bottom row shows NKT-like cell subset response. CD3^+^ CD56^+^ CD8^+^ and CD3^+^ CD56^+^ CD57^+^ are expressed as % of NKT cells. CD3^+^ CD56^+^ CD8^+^ CD57^+^ are expressed as % of NKT CD8^+^ cells.

The main lymphocyte populations i.e., NK cells, T-helper cells, cytotoxic T cells and B cells (CD3^-^CD19^+^) remained unchanged over the study year, both in absolute numbers (cells/µL) and proportions (% of lymphocytes) (Supplementary Table S12). However, several sub populations were significantly and consistently altered in association with the intervention. Within the NK cell population (Fig. 1), there was a significant reduction in pro-inflammatory NKG2C expressing NK cells (CD3^−^CD56^dim^NKG2C^+^NKG2A^−^, P = 0.0003), and a significant increase in reciprocal anti-inflammatory NKG2A expressing NK cells (CD3^−^CD56^dim^NKG2C^−^NKG2A^+^, P = 0.032). Simultaneously, there was a significant reduction in NK cells expressing CD57, which identifies terminally differentiated, senescent cells. Regarding NKT-like cells, the total population of CD3^+^CD56^+^ cells increased from 9.1 to 10.5%, P = 0.00001 (Supplementary Table S13). In contrast, the subpopulation of CD8^+^ NKT-like cells decreased and so did the populations of terminally differentiated CD57^+^ cells, both within the total NKT-like population and the CD8^+^ NKT-like population (Fig. 1). Significant results for subpopulations of NK cells and NKT-like cells are shown in Fig. 1 for visualisation of individual changes. Complete data for all NK and NKT-like subsets are presented in Supplementary Table S13.

For T helper cells there was a significant reduction in T_CM_ (P = 1.09 × 10^−8^) and a significant increase in naïve T cells (P = 0.0008) and T_EM,_ (P = 0.006). Among cytotoxic T cells there was also a decrease in central memory cells (CD8^+^T_CM,_ P = 0.008) while CD8^+^T_EMRA_ increased (P = 0.005). There was no significant change in regulatory T cells (Treg cells; CD25^+^Foxp3^+^). All results for CD4^+^ and CD8^+^ populations are shown in Table 4.

**Table 4:** Subpopulations of T-helper cells and cytotoxic T cells at baseline and follow-up.

		Baseline	Follow-up	Difference	*p*
**T helper cells**	CD3^+^CD4^+^				
Naïve^a^	*CD62L*^+^*CD45RA*^+^	41.6 (± 13.8)	43.4 (± 14.2)	+ 1.7 (± 3.0)	0.0008
Central memory^a^	*CD62L*^+^*CD45RA-*	41.4 (± 10.3)	38.6 (± 10.0)	− 2.8 (± 2.5)	1.087 × 10^−8^
Effector memory^a^	*CD62L-CD45RA-*	14.3 (± 7.05)	15.3 (± 7.75)	+ 1.0 (± 2.2)	0.006
TEMRA^a^	*CD62L-CD45RA*^+^	2.62 (± 3.37)	2.78 (± 3.56)	+ 0.2 (± 1.2)	0.373
Activated	*HLA-DR*^+^	8.1 (± 3.46)	7.0 (± 3.83)	− 1.1 (± 2.4)	0.002
Senescent T helper cells	*CD28-*	6.52 (± 10.1)	6.15 (± 7.11)	− 0.4 (± 6.2)	0.678
Non-senescent T helper cells	*CD28*^+^	93.5 (± 10.1)	93.9 (± 7.12)	+ 0.4 (± 6.2)	0.685
Regulatory T cells	*CD25*^+^*Foxp3*^+^	7.07 (± 1.80)	6.83 (± 1.72)	− 0.2 (± 1.1)	0.126
**Cytotoxic T cells**	CD3^+^CD8^+^				
Naïve	*CD62L*^+^*CD45RA*^+^	26.5 (± 11.5)	26.0 (± 10.8)	− 0.5 (± 3.6)	0.434
Central memory	*CD62L*^+^*CD45RA-*	18.7 (± 9.58)	17.4 (± 9.17)	− 1.3 (± 2.9)	0.008
Effector memory cells	*CD62L*^*-*^*CD45RA-*	18.9 (± 9.28)	18.2 (± 9.07)	− 0.7 (± 2.7)	0.132
TEMRA	*CD62L*^*-*^*CD45RA*^+^	35.9 (± 16.4)	38.3 (± 16.3)	+ 2.4 (± 5.1)	0.005
Activated	*HLA-DR*^+^	14.5 (± 10.8)	14.1(± 11.0)	− 0.4 (± 5.4)	0.598
Senescent cytotoxic T cells	*CD28-*	45.6 (± 20.6)	45.6 (± 22.2)	− 0.1 (± 6.8)	0.942
Non-senescent cytotoxic T cells	*CD28*^+^	54.4 (± 20.6)	54.4 (± 22.2)	+ 0.1 (± 6.8)	0.939

*TEMRA* Terminally differentiated effector memory cells.

All subpopulations are expressed as proportion (%) of CD4^+^ or CD8^+^ cells.

The difference refers to the absolute difference (the follow-up level subtracted from the baseline level).

^a^Due to technical issues of the CD45RA antibody, results from nine patients were excluded from the analysis of naïve, central memory, effector memory and TEMRA in CD4^+^ and CD8^+^ cells. Additionally two patients were identified with a genetic variation causing inability to downregulate CD45RA in effector cells^21^ and were therefore excluded from analyses based on CD45RA expression. This resulted in in data from 39 patients being used for populations based on CD45RA, and data from 50 patients in the remaining analyses in this table.

### Myeloid cells increased in numbers

As shown in Table 3, there were significant changes in the myeloid cell compartment. There was an increase in monocytes, basophils, and neutrophils during the study year.

### Plasma protein profiling

Of 176 proteins that were analysed, 159 were detectable in more than 70% of the samples and these were included in further analyses. There were significant changes in 16 proteins, with the most prominent changes in TNF-Related Apoptosis Inducing Ligand (TRAIL) (P = 0.001), Heat Shock 27 kDa Protein (HSP27) (P = 0.003) and Follistatin (FS) (P = 0.005) (Supplementary Table 14).

### Functional annotation and protein interactions

**Figure 2:**
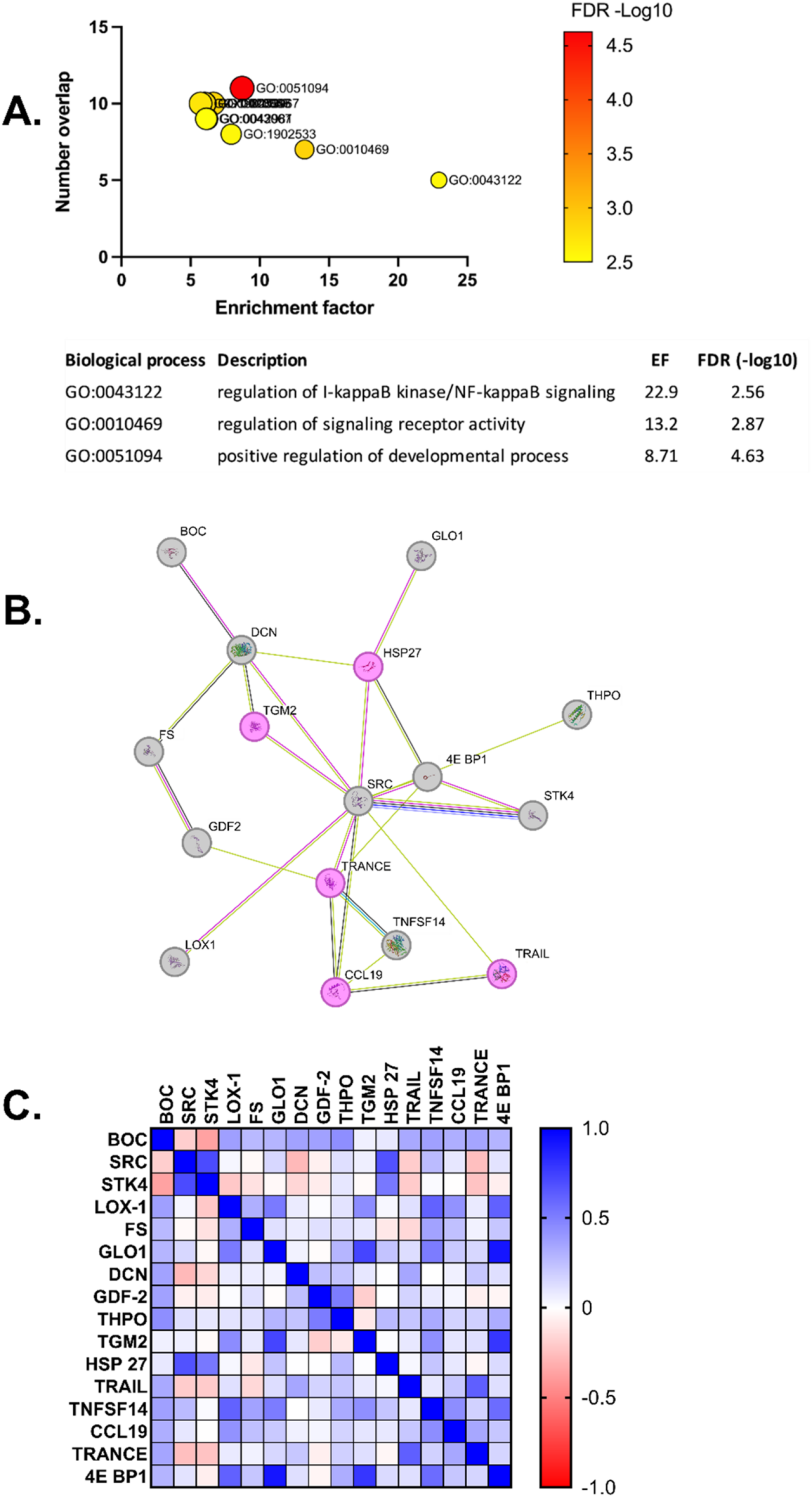
Prediction of biological significance of differently expressed proteins in patients undergoing cardiovascular risk management. Over-representation analysis (ORA) of 16 differently expressed plasma proteins in biological processes (BP) before and after cardiovascular risk-management using WebGestalt analysing tool. (**A**) Five to eleven proteins were over-represented in 10 BP with enrichment factors (EF) between 5.7 and 23 in combination with false discovery rate (FDR) < 0.01. Size of symbols correlates with the number of proteins and colour of symbols with FDR. (**B**) Predicted protein interactions of the differently expressed proteins by the String database vs 11.5, confidence score > 0.250. Pink indicates proteins over-represented in the BP I-κB kinase/NF-κB signalling. Black lines indicate co-expression of proteins, pink lines indicate experimentally determined interaction and green lines interactions based on text mining. (**C**) Correlation-matrix of the factors included in the predicted protein interaction plot. The genes/proteins included are further described in Supplementary Table S14.

We sought to identify biologically meaningful information from the differently expressed plasma proteins. Over-representation analysis of the 16 plasma proteins revealed a significant overlap (FDR < 0.01) of ten different gene ontology (GO) terms (Fig. 2A). Among the differently expressed proteins, five proteins related to I-κB kinase/NF-κB signalling were most enriched (FDR = 0.003 and enrichment ratio 22.9). These included Tumour necrosis factor ligand superfamily member 11 (TRANCE), Heat Shock Protein Family B Member 1 (HSPB1), Protein-glutamine gamma-glutamyltransferase 2 (TGM2), TNF Superfamily Member 10 (TNFSF10) and C–C motif chemokine 19 (CCL19) (Fig. 2A–C). A protein interaction network was generated using the STRING v11.5 (http://string-db.org/) database to identify potential binding partners for proteins involved in NF-κB signalling. Known and predicted protein interactions were revealed between four of the proteins linked to NF-κB signalling and eight other differentially expressed proteins (Fig. 2B). The relevant proteins shown as a correlation matrix in Fig. 2C with further details provided in Supplementary Table S14. Also, String analysis confirmed the over-representation of proteins associated with the NF-κB signalling pathway (FDR = 0.01).

## Discussion

We show that cardiovascular risk management in patients with advanced atherosclerotic lesions increases the proportions of naïve cells in several inflammatory cell populations. For the first time, this study explores sub lineages of NK cells, NKT-like cells, and T cells and how they respond to cardiovascular risk management. The major lymphocyte populations (T, B and NK cells) remained unchanged, and no strong linear correlations were observed between outcome measures and specific cell populations. However, cardiovascular risk management was associated with several consistent findings in sub-lineages of these cells.

We observed a significant reduction in NKG2C^+^ NK cells, and a significant increase in NKG2A^+^ NK cells. Previously, NK cells have been shown to acquire anti-inflammatory properties through NKG2A, while the blockage of NKG2A aggravates neutrophil-induced inflammation^27^. Thus, our findings are consistent with an altered balance between cell subsets, possibly indicating a shift towards a more anti-inflammatory NK cell phenotype in response to cardiovascular risk management. In accordance, there was also a decrease in terminally differentiated (CD57^+^) cells both in the NK cell and NKT-like cell lineages. CD57 is looked upon as a marker for NK cells with poor proliferative- and cytotoxic capacity^28,29^. Historically CD57 has been associated with immune senescence, but at a closer look, progression from CD56^bright^ to CD56^dim^CD57^−^ to CD56^dim^CD57^+^ might reflect a maturation pathway rather towards a higher cytotoxic capacity and decreased responsiveness to cytokines, and not a true senescent state^28^. In the general population, CD57 expression increases with age and with exposure to infections, contrary to the tendency seen in our study cohort^29^. Similarly, in CD4^+^ cells there was a significant reduction in central memory cells and a significant increase in naïve T cells. As we only assessed the proportions of subpopulations, and not absolute counts, we cannot determine whether the more naïve profile stems from an increased amount of naïve cells or a decreased amount of more mature phenotypes in the circulation, e.g. through apoptosis of more mature populations or increased proliferation of naïve cells.

It is well established that T cell differentiation induces T cell aging. With time and upon stimulation, naïve T cells differentiate to memory cells or effector T cells. Finally, they evolve into terminally differentiated T effector memory cells that re-express CD45RA (T_EMRA_), These are regarded as exhausted T cells, although recent data show their heterogeneity and a likely anti-viral role for subpopulations of these cells^30^. Importantly, senescent T cells produce an excessive amount of pro-inflammatory cytokines^31^. Therefore, the shift towards less differentiated CD4^+^ cells may be indicative of a reduction in chronic inflammation with potential anti-atherosclerotic effects. The functional properties of terminally differentiated NK cells are less well established^28^ and it is less clear if the transition to less differentiated NK cells as seen here, may have anti-inflammatory effects. Taken together, the biological significance of the shift towards less differentiated T cell, NK cell and NKT-like populations needs to be established in future studies.

We did not see a decrease in neutrophil levels. Instead, a statistically significant increase was observed that was paralleled by a significant increase in the neutrophil:leukocyte ratio, and increased numbers of other cells of myeloid lineage i.e. monocytes and basophils. Elevated neutrophil counts and neutrophil/lymphocyte ratios are indicative of the presence and severity of atherosclerotic disease and statin treatment has been shown to reduce neutrophil counts^32–34^. The reason for the increase in neutrophils in our study is unclear. Speculatively, subsets of neutrophils might be more sensitive than the total neutrophil population to the moderate changes in cardiovascular risk factors observed in our study. Many of the receptors that activate NF-κB pathways are expressed on myeloid cell types, suggesting that NF-κB might play an important role in their development and activation^35^. However, our protein profiling data and their functional annotation only demonstrate weak indications that this pathway responds to optimized cardiovascular risk management and therefore no firm conclusions can be drawn regarding the cause for the unexpected increase in neutrophils, monocytes and basophils. Possibly, to reveal how myeloid cells respond to cardiovascular prevention treatment, subsets of these cells should be studied.

The effect of statin treatment on T cells has been studied previously. For example, statin treatment has been shown to reduce levels of senescent T helper cells (CD4^+^CD28^−^)^15^. This effect was not observed in our study where CD28^−^ senescent T helper cells remained unchanged. However, the data on statin treatment and CD4^+^CD28^−^ T cells is not uniform, and responses might differ between stable and unstable stages of the atherosclerotic disease^36^. Tregs are considered to have anti-atherogenic properties and previous studies report an increase in numbers of CD4^+^CD25^+^FOXP3^+^ T cells upon statin treatment^37–39^. Contrary to these earlier findings, we did not see a significant change in Treg levels. A possible explanation for the stable levels of both Treg and CD28 expressing cells could be that in most of the patients who participated in our study, statin treatment was initiated years before the start of the study, albeit at suboptimal dosages. Therefore, these changes might have occurred before the patients entered the study.

Statin effects on NK cells have been reported previously, but only with respect to the total NK population. Previous studies found no significant differences in NK cell counts in response to statin treatment^40,41^. In the current study, for the first time, NK cell subsets were analysed, and significant changes were observed in the activation/differentiation state of these subsets. Thus, our findings emphasize the need to analyse NK subsets to understand the NK cell response to cardiovascular risk management.

We achieved significant reductions in LDL but there was no linear relation between LDL reduction and the changes in immune cell populations. Most patients were already on statin treatment before study inclusion, but patients were undertreated. Accordingly, lipid lowering treatment was adjusted in the majority of patients. Therefore, one might reason that, the changes we observed might be driven in part by the pleiotropic i.e., cholesterol-independent effects of lipid lowering treatment. For example, statin mediated inhibition of isoprenoid intermediates during the biosynthesis of cholesterol is shown to inhibit proteins like Ras, Rho, and Rac (known regulators of cellular differentiation, apoptosis, and cell proliferation) resulting in the inhibition of enzymes like Rho kinase (ROCK). The inhibition of ROCK, reduces intimal hyperplasia and increases nitric oxide production in the endothelium^42^. Further, statins are known to improve endothelial function also through the subdued release of free radicals, decreased synthesis of endothelin-1 and upregulation of endothelial progenitor cells^43^. Ezetimibe, which was also prescribed within the context of this study, has not been shown to reduce CRP when used as monotherapy^44^. Thus, it can be argued that ezetimibe does not reduce global inflammation. However, ezetimibe has been shown to suppress NF-κB activation via the mitogen-activated protein kinase pathway, implying another type of immune interference^45^. These are examples of cholesterol independent mechanisms of statins and ezetimibe that have potential implications for immune cell differentiation and may explain immunological responses to cardiovascular risk management to some degree. However, as of yet these mechanisms have been insufficiently studied in this regard and the mechanistic reasons for the changes in NK and T cell subpopulations seen here, remain to be explored in future studies.

In contrast to CD4^+^ lymphocytes, there was no increase in the number of less differentiated CD8^+^ cells, but instead an increase in the T_EMRA_-population. Prior cytomegalovirus (CMV) infection affects the senescence of naïve CD8^+^ lymphocytes and most patients had elevated antibody titres against CMV. Accordingly, CMV positive patients showed lower levels of naïve CD8^+^ T cells and higher levels of CD8^+^ T_EMRA_ cells compared to CMV negative patients. This is in line with previous studies which show that CMV drives T cells towards a later stage of differentiation^46^. CMV dramatically modulates the overall immune profile of CD8^+^ T-cells, and this is likely to overshadow any interventional effect from cardiovascular risk management^47^. Interestingly, there is a documented CMV-mediated effect on mature subpopulations in the CD4^+^ compartment as well^48^. The effect is not as attenuated as for CD8^+^ cells but may explain the observed increase in CD4^+^ T_EM_.

Immune cell phenotypes exhibit a natural variation with a decrease in naïve cells over time. Theoretically, this could be a confounding factor contributing to changes in lymphocyte subsets. However, this is unlikely because we show a relative increase in naïve phenotypes which is opposite to the increase in senescent cells seen normally with the passage of time. Furthermore, longitudinal studies have shown that lymphocyte phenotypes are stable over time^49^.

Although CRP participates in atherogenesis, we did not find any strong correlation between hsCRP and the cellular changes observed in the current study^50^. The only statistically significant correlations were between hsCRP and CD3^+^ and CD4^+^CD8^+^ cells, but these were weak. In our interpretation these results were not indicative of any mechanistic relationship between the change in hsCRP and the changes in CD3^+^ and CD4^+^8^+^ cells. The acute-phase reactant CRP serves as a pattern-recognition molecule in the innate immune system and its main biological function is a first-line innate host defense^50^. CD3^+^ and CD4^+^CD8^+^ cells participate in adaptive immunity, and this possibly explains the lack of a strong correlation between hsCRP levels and the changes in these two cell populations.

Flow cytometric analysis was complemented by plasma protein profiling. At follow-up 16 proteins were differentially expressed, compared to baseline. Five of these proteins were associated with NF-κB signalling. NF-κB coordinates multiple processes in atherosclerotic plaque development, and previous in vitro studies have shown that statin treatment reduces NF-κB activation^51^ in vascular smooth cells and mononuclear cells^51^. However, the net effect on NF-κB signalling cannot be determined from the current data since directionality and causality cannot be assessed from correlations derived from curated databases. In addition, the cytokines dependent on NF-κB signalling that were measured in the current study (IL-6, TNF, and chemokine CCL2) were unchanged. This might indicate that cardiovascular risk management does not affect NF-κB signalling. However, other cytokines dependent on NF-κB, for example VCAM-1, ICAM-1 and M-CSF were not measured. Therefore, a potential change in NF-κB signalling may have been missed^52^. Taken together, the effects of cardiovascular risk management on NF-κB signaling are not fully understood and merit further investigation.

We only achieved moderate changes in the cardiovascular risk profile of the study participants. Guidelines recommend multi-disciplinary cardiovascular risk management teams. However, in Sweden these teams are not yet available for patients with carotid atherosclerosis. The lack of a multidisciplinary team with specialist nurses, physical therapists, dieticians and psychosocial councillors may have contributed to the absence of more pronounced effects on the outcome variables. It is well established that the ability to adopt a healthy lifestyle is influenced by the impact of a diagnosis or symptoms, degree of education, socioeconomic factors, cognitive and emotional factors, and mental health. Thus, the degree of non-adherence to medication and lifestyle advice seen in the current study might have been reduced if patients had access to a multidisciplinary team where the knowledge and skills of different caregivers may be combined in multidisciplinary behavioural approaches, as recommended by current guidelines^1^. Statin intolerance further contributed to non-adherence to medication. At the time of the study PCSK9-inhibitors were not yet available in Sweden. Thus, it is unclear if our patients would have benefitted from a switch to these alternatives for statin treatment. Furthermore, high grade carotid stenosis represents an advanced stage of atherosclerotic disease. The patient group in this study is therefore elderly with concomitant diseases, creating impediments for optimal prevention treatment that might be more pronounced than in a relatively younger population, for example those who have experienced their first myocardial infarction. Kidney function deteriorated during the study year. Apart from atherosclerosis the majority of patients had hypertension and a sizable number had diabetes, thus a slow progression of nephropathy might be expected in the current population. Furthermore, the prescription of ACE-inhibitors and diuretics increased. Although this increase did not reach statistical significance, it may have contributed to the deterioration of kidney function.

We did not include a control group, and this is a limitation of our study. However, patients with advanced carotid atherosclerosis rank in the very highest risk category for future cardiovascular events and we considered it unethical to leave patients without treatment solely for the purpose of the current study.

In conclusion, cardiovascular risk management results in a consistent increase in more naïve phenotypes among NK, NKT-like and CD4^+^ T cells, with a concomitant decrease of senescent phenotypes, suggesting a transition towards a less pro-inflammatory lymphocyte profile in response to cardiovascular risk management. This indicates a shift away from the senescent, pro-inflammatory immune cell phenotype seen in atherosclerosis. The clinical and biological relevance of these findings remain to be established, but it is encouraging that cardiovascular risk management appears to exhibit advantageous immunological effects.

### Supplementary Information

Supplementary Information.

## Data Availability

The datasets generated during and/or analysed during the current study are available from the corresponding author on reasonable request.
