# Supplementary material for: Changes in natural killer and T lymphocyte phenotypes in response to cardiovascular risk management
Source: Sci Rep. 2023 Nov 27;13:20810. doi: 10.1038/s41598-023-48111-7 (PMC10682417; doi:10.1038/s41598-023-48111-7)
Supplement: Supplementary file 1 — Supplementary Information. [file 41598_2023_48111_MOESM1_ESM.pdf]

Supplementary Tables and Figures

Supplementary Table S1.

A. Monoclonal antibodies and reagents

Antibody	Clone	Manufacturer
CD45RA-FITC	L48	BD Biosciences
CD28 PE	L293	BD Biosciences
HLA-DR- PerCP	L243	BD Biosciences
CD4 PE-Cy7	SK3	BD Biosciences
CD62L- APC	Dreg56	BD Biosciences
CD8- APC-H7	SK1	BD Biosciences
CD3- Horizon V450	UCHT1	BD Biosciences
CD57- FITC	HNK-1	BD Biosciences
CD85j-PE, LIR-1	GHI/75	BD Biosciences
CD56- PE-Cy7	NCAM16.2	BD Biosciences
CD4-PerCp	SK3	BD Biosciences
CD25-PE-Cy7	2A3	BD Biosciences
CD152-APC, CTLA-4	BNI3	BD Biosciences
NKG2C-PerCP	134591	R&D
CD159a-APC, NKG2A	PNA60797	Beckman Coulter
Foxp3-PE	PCH101	eBioscience
**Multitest reagent**	**Description**	**Manufacturer**
340491 for analysis of T cells	FITC-CD3, PE-CD8, PerCP-CD45 and APC-CD4	BD Biosciences
342417 for analysis of B- and NK cells	FITC-CD3, PE-CD16 and CD56, PerCP-CD45 and APC-CD19	BD Biosciences

B. Flow cytometry panels for T- and NK cell subsets

	FITC	PE	PerCP	PE-Cy7	APC	APC-H7	H V450
T cell panel	CD45RA	CD28	HLA-DR	CD4	CD62L	CD8	CD3
NK cell panel	CD57	CD85j (LIR-1)	NKG2C	CD56	NKG2A (CD159a)	CD8	CD3
Treg panel	CD45RA	Foxp3	CD4	CD25	CTLA-4 (CD152)		CD3

Supplementary Table S2. Protein assay list, Olink Target 96 Cardiovascular II, v.5007

2,4-dienoyl-CoA reductase, mitochondrial	Q16698	Gastric intrinsic factor (GIF)	P27352
A disintegrin and metalloproteinase with thrombospondin motifs 13 (ADAM-TS13)	Q76LX8	Gastrotropin (GT)	P51161
ADM (ADM)	P35318	Growth hormone (GH)	P01241
Agouti-related protein (AGRP)	O00253	Growth/differentiation factor 2 (GDF-2)	Q9UK05
Alpha-L-iduronidase (IDUA)	P35475	Heat shock 27 kDa protein (HSP 27)	P04792
Angiopoietin-1 (ANG-1)	Q15389	Heme oxygenase 1 (HO-1)	P09601
Angiopoietin-1 receptor (TIE2)	Q02763	Hydroxyacid oxidase 1 (HAOX1)	Q9UJM8
Angiotensin-converting enzyme 2 (ACE2)	Q9BYF1	Interleukin-1 receptor antagonist protein (IL-1ra)	P18510
Bone morphogenetic protein 6 (BMP-6)	P22004	Interleukin-1 receptor-like 2 (IL1RL2)	Q9HB29
Brother of CDO (Protein BOC)	Q9BWV1	Interleukin-4 receptor subunit alpha (IL-4RA)	P24394
Carbonic anhydrase 5A, mitochondrial (CA5A)	P35218	Interleukin-6 (IL6)	P05231
Carcinoembryonic antigen related cell adhesion molecule 8 (CEACAM8)	P31997	Interleukin-17D (IL-17D)	Q8TAD2
Cathepsin L1 (CTSL1)	P07711	Interleukin-18 (IL-18)	Q14116
C-C motif chemokine 3 (CCL3)	P10147	Interleukin-27 (IL-27)	Q8NEV9, Q14213
C-C motif chemokine 17 (CCL17)	Q92583	Kidney Injury Molecule (KIM1)	Q96D42
CD40 ligand (CD40-L)	P29965	Lactoylglutathione lyase (GLO1)	Q04760
Fatty acid-binding protein, intestinal (FABP2)	P12104	Lymphotactin (XCL1)	P47992
Fibroblast growth factor 21 (FGF-21)	Q9NSA1	Macrophage receptor MARCO (MARCO)	Q9UEW3
Fibroblast growth factor 23 (FGF-23)	Q9GZV9	Matrix metalloproteinase-7 (MMP-7)	P09237
Follistatin (FS)	P19883	Matrix metalloproteinase-12 (MMP-12)	P39900
Galectin-9 (Gal-9)	O00182	Melusin (ITGB1BP2)	Q9UKP3
Natriuretic peptides B (BNP)	P16860	Serine protease 27 (PRSS27)	Q9BQR3
NF-κ-B essential modulator (NEMO)	Q9Y6K9	Serine/threonine-protein kinase 4 (STK4)	Q13043
Osteoclast-associated immunoglobulin-like receptor (hOSCAR)	Q8IYS5	Serpin A12 (SERPINA12)	Q8IW75
Pappalysin-1 (PAPPA)	Q13219	SLAM family member 5 (CD84)	Q9UIB8
Pentraxin-related protein PTX3 (PTX3)	P26022	SLAM family member 7 (SLAMF7)	Q9NQ25
Placenta growth factor (PGF)	P49763	Sortilin (SORT1)	Q99523
Platelet-derived growth factor subunit B (PDGF subunit B)	P01127	Spondin-2 (SPON2)	Q9BUD6
Poly [ADP-ribose] polymerase 1 (PARP-1)	P09874	Stem cell factor (SCF)	P21583
Polymeric immunoglobulin receptor (PIgR)	P01833	Superoxide dismutase [Mn], mitochondrial (SOD2)	P04179
Programmed cell death 1 ligand 2 (PD-L2)	Q9BQ51	T-cell surface glycoprotein CD4 (CD4)	P01730
Proheparin-binding EGF-like growth factor (HB-EGF)	Q99075	Thrombomodulin TM	P07204
Pro-interleukin-16 (IL16)	Q14005	Thrombopoietin (THPO)	P40225
Prolargin (PRELP)	P51888	Thrombospondin-2 (THBS2)	P35442

Prostasin (PRSS8)	Q16651	Tissue factor (TF)	P13726
Protein AMBP (AMBP)	P02760	TNF-related apoptosis-inducing ligand receptor 2 (TRAIL-R2)	O14763
Proteinase-activated receptor 1 (PAR-1)	P25116	Tumor necrosis factor receptor superfamily member 10A (TNFRSF10A)	O00220
Protein-glutamine gamma-glutamyltransferase 2 (TGM2)	P21980	Tumor necrosis factor receptor superfamily member 11A (TNFRSF11A)	Q9Y6Q6
Proto-oncogene tyrosine-protein kinase Src (SRC)	P12931	Tumor necrosis factor receptor superfamily member 13B (TNFRSF13B)	O14836
P-selectin glycoprotein ligand 1 (PSGL-1)	Q14242	Tyrosine-protein kinase Mer (MERTK)	Q12866
Receptor for advanced glycosylation end products (RAGE)	Q15109	Vascular endothelial growth factor D (VEGFD)	O43915
Renin (REN)	P00797	V-set and immunoglobulin domain-containing protein 2 (VSIG2)	Q96IQ7

Supplementary Table S3. Protein assay list, Olink Target 96 Inflammation, v.3023

Adenosine Deaminase (ADA)	P00813	Fibroblast growth factor 23 (FGF-23)	Q9GZV9
Artemin (ARTN)	Q5T4W7	Fibroblast growth factor 5 (FGF-5)	Q8NF90
Axin-1 (AXIN1)	O15169	Fibroblast growth factor 19 (FGF-19)	O95750
C-C motif chemokine 23 (CCL23)	P55773	Interleukin-2 (IL-2)	P60568
C-C motif chemokine 25 (CCL25)	O15444	Interleukin-2 receptor subunit beta (IL-2RB)	P14784
C-C motif chemokine 28 (CCL28)	Q9NRJ3	Interleukin-4 (IL-4)	P05112
CD40L receptor (CD40)	P25942	Interleukin-5 (IL5)	P05113
CUB domain-containing protein 1 (CDCP1)	Q9H5V8	Interleukin-6 (IL6)	P05231
C-X-C motif chemokine 1 (CXCL1)	P09341	Interleukin-7 (IL-7)	P13232
C-X-C motif chemokine 5 (CXCL5)	P42830	Interleukin-8 (IL-8)	P10145
C-X-C motif chemokine 6 (CXCL6)	P80162	Interleukin-10 (IL10)	P22301
C-X-C motif chemokine 9 (CXCL9)	Q07325	Interleukin-10 receptor subunit alpha (IL-10RA)	Q13651
C-X-C motif chemokine 10 (CXCL10)	P02778	Interleukin-10 receptor subunit beta (IL-10RB)	Q08334
C-X-C motif chemokine 11 (CXCL11)	O14625	Interleukin-12 subunit beta (IL-12B)	P29460
Cystatin D (CST5)	P28325	Interleukin-13 (IL-13)	P35225
Delta and Notch-like epidermal growth factor-related receptor (DNER)	Q8NFT8	Interleukin-15 receptor subunit alpha (IL-15RA)	Q13261
Eotaxin (CCL11)	P51671	Interleukin-17A (IL-17A)	Q16552
Eukaryotic translation initiation factor 4E-binding protein 1 (4E-BP1)	Q13541	Interleukin-17C (IL-17C)	Q9P0M4
Fibroblast growth factor 21 (FGF-21)	Q9NSA1	Interleukin-18 (IL-18)	Q14116
Interleukin-18 receptor 1 (IL-18R1)	Q13478	Programmed cell death 1 ligand 1 (PD-L1)	Q9NZQ7
Interleukin-20 (IL-20)	Q9NYY1	Protein S100-A12 (EN-RAGE)	P80511
Interleukin-24 (IL-24)	Q13007	SIR2-like protein 2 (SIRT2)	Q8IXJ6
Interleukin-33 (IL-33)	O95760	STAM-binding protein (STAMBP)	O95630
Latency-associated peptide transforming growth factor beta-1 (LAP TGF-beta-1)	P01137	Stem cell factor (SCF)	P21583
Leukemia inhibitory factor (LIF)	P15018	Sulfotransferase 1A1 (ST1A1)	P50225
Leukemia inhibitory factor receptor (LIF-R)	P42702	T cell surface glycoprotein CD6 isoform (CD6)	Q8WWJ7
Macrophage colony-stimulating factor 1 (CSF-1)	P09603	T-cell surface glycoprotein CD5 (CD5)	P06127
Matrix metalloproteinase-1 (MMP-1)	P03956	T-cell surface glycoprotein CD8 alpha chain (CD8A)	P01732
Matrix metalloproteinase-10 (MMP-10)	P09238	Thymic stromal lymphopoietin (TSLP)	Q969D9
Monocyte chemotactic protein 1 (MCP-1)	P13500	TNF-beta (TNFB)	P01374
Monocyte chemotactic protein 2 (MCP-2)	P80075	TNF-related activation-induced cytokine (TRANCE)	O14788
Monocyte chemotactic protein 3 (MCP-3)	P80098	TNF-related apoptosis-inducing ligand (TRAIL)	P50591
Monocyte chemotactic protein 4 (MCP-4)	Q99616	Transforming growth factor alpha (TGF-alpha)	P01135

Natural killer cell receptor 2B4 (CD244)	Q9BZW8	Tumour necrosis factor (Ligand) superfamily, member 12 (TWEAK)	O43508
Neurotrophin-3 (NT-3)	P20783	Tumour necrosis factor (TNF)	P01375
Neurturin (NRTN)	Q99748	Tumour necrosis factor ligand superfamily member 14 (TNFSF14)	O43557
Oncostatin-M (OSM)	P13725	Tumour necrosis factor receptor superfamily member 9 (TNFRSF9)	Q07011
Osteoprotegerin (OPG)	O00300	Urokinase-type plasminogen activator (uPA)	P00749
		Vascular endothelial growth factor A (VEGF-A)	P15692

Supplementary Table S4. Cardiovascular risk factors at baseline

Patient risk factors (N, %)	
LDL ≥1.8	33 (66.0)
Body Mass Index >25	42 (84)
Waist >102 cm for men, > 88 cm for women	30 (60.0)
Diabetes	12 (24.0)
Hypertension	42 (84.0)
Current smoking	7 (14.0)
Previous smoking	29 (58.0)
Alcohol overconsumption[1]	0 (0)
Angina or MI in a 1st degree relative <60y	10 (20.0)
No- or low level of exercise	37 (74.0)
Poor diet[2]	5 (10.0)

N= Number of patients

Cardiovascular risk factors. Baseline analysis of the 50 patients in the study.
[1] >15 units per week for men, >9 units per week for women, limits set according to guidelines at time for the study[25].
[2] Based on questionnaire from the Swedish Board of Health

Supplementary Table S5. CMV subgroup analysis showing lymphocyte quantities over the study year comparing CMV negative and CMV positive subjects.

		**CMV status (IgG)**	**Baseline** Mean	**Baseline** SD	**F**	**P**	**Follow-up** Mean	SD	**F**	**P**	**Difference** Mean	SD	**F**	**P**
Leukocytes	*x10⁹/L*	Negative	7.35	±1.70	0.41	0.526	7.39	±1.70	0.08	0.776	0.05	±0.71	0.343	0.561
		Positive	7.74	±1.83			7.55	±1.62			-0.19	±1.26		
Lymphocytes	*x10⁹/L*	Negative	1.69	±0.38	1.13	0.293	1,55	±0.28	5.92	0.019	-0.14	±0.34	3.137	0.083
		Positive	1.86	±0.48			1,93	±0.48			0.07	±0.34		
T-cells	*x10⁶/L*	Negative	1165	±326	3.14	0.083	1129	±263	6.12	0.017	-35.91	±213	0.812	0.372
		Positive	1405	±414			1449	±404			43.74	±270		
T_H cells	*x10⁶/L*	Negative	845	±265	0.78	0.382	821	±233	2.06	0.158	-24.73	±136	0.433	0.514
		Positive	931	±290			943	±253			11.59	±168		
T_C cells	*x10⁶/L*	Negative	275	±163	7.59	0.008	260	±174	9.70	0.003	-14.27	±79.8	1.313	0.258
		Positive	475	±224			506	±244			31.33	±124		
B-cells	*x10⁶/L*	Negative	160	±53.3	0.56	0.456	154	±52.4	0.70	0.406	-5.36	±27.8	0.152	0.699
		Positive	180	±85.9			180	±95.5			-0.69	±36.8		
NK-cells	*x10⁶/L*	Negative	359	±130	3.31	0.075	262	±82.5	0.15	0.696	-97.82	±137	12.154	0.001
		Positive	267	±154			285	±196			18.85	±84.8		

Mean lymphocyte levels in CMV negative (N=11) and CMV positive (N=39) individuals at baseline and follow-up. One-way ANOVA was used to assess leukocyte response over the study year and potential differences between groups.

Supplementary Table S6. CMV subgroup analysis showing NK cell quantities over the study year comparing CMV negative and CMV positive subjects.

Population		CD markers	CMV status (IgG)	Baseline Mean	SD	F	P	Follow-up Mean	SD	F	P	Difference Mean	SD	F	P
Total NK cells	*% of Ly*	*CD3-CD56$^+$*	Negative	18.75	±7.11	6.20	0.016	16.18	±5.39	0.90	0.348	-2.56	±4.46	11.4	0.001
			Positive	12.97	±6.70			13.84	±7.65			0.87	±2.45		
Immature	*% of NK*	*CD56^{bright}*	Negative	3.22	±0.81	3.27	0.077	4.01	±1.71	1.00	0.322	0.79	±1.51	3.69	0.061
			Positive	5.05	±3.32			5.03	±3.25			-0.02	±1.16		
Mature	*% of NK*	*CD56^{dim}*	Negative	96.78	±0.81	3.28	0.076	95.99	±1.71	1.00	0.321	-0.79	±1.51	3.71	0.060
			Positive	94.94	±3.32			94.97	±3.25			0.02	±1.16		
-Differentiated	*% of Mature NK*	*CD57$^+$*	Negative	44.58	±17.32	0.87	0.355	38.21	±14.61	1.86	0.179	-6.37	±5.04	2.85	0.098
			Positive	50.46	±18.73			46.60	±18.82			-3.86	±4.15		
-KIR	*% of Mature NK*	*CD85j$^+$*	Negative	32.74	±14.05	6.62	0.013	33.79	±12.69	4.59	0.037	1.05	±3.05	3.03	0.088
			Positive	44.72	±13.53			43.97	±14.21			-0.75	±3.03		
-Activated	*% of Mature NK*	*NKG2C$^+$*	Negative	2.96	±1.26	1.48	0.230	1.58	±1.12	0.84	0.363	-1.38	±1.19	0.40	0.530
			Positive	6.51	±9.60			3.93	±8.42			-2.15	±3.94		
-Resting	*% of Mature NK*	*NKG2A$^+$*	Negative	45.07	±12.81	2.98	0.091	46.22	±13.48	2.34	0.132	1.15	±3.49	0.03	0.858
			Positive	37.40	±13.03			39.05	±13.72			0.95	±3.06		
NKT-like cells	*% of Ly*	*CD3$^+$CD56$^+$*	Negative	6.27	±4.93	5.38	0.025	8.12	±4.47	2.64	0.111	1.85	±1.52	0.84	0.365
			Positive	10.02	±4.68			11.12	±5.63			1.10	±2.56		
-Differentiated	*% of NKT-like*	*CD3$^+$CD56$^+$ CD57$^+$*	Negative	33.60	±25.33	17.3	0.000	21.71	±17.42	29.2	0.000	-11.9	±9.05	2.69	0.108
			Positive	60.94	±17.33			54.54	±17.90			-6.40	±9.99		

Mean difference between baseline and follow-up, comparing the CMV negative group (N=11) with the positive group (N=39).

Supplementary Table S7. CMV subgroup analysis showing quantities of T-helper cells and cytotoxic T cells over the study year comparing CMV negative and CMV positive subjects.

		**CMV status (IgG)**	**Baseline** Mean	SD	***F***	**P**	**Follow-up** Mean	SD	***F***	**P**	**Difference** Mean	SD	***F***	**P**
T helper cells	***$CD3^+CD4^+$***	Negative	46.8	±8.09	0.03	0.868	50.0	±12.3	0.70	0.405	+3.26	±6.96	2.32	0.135
		Positive	47.2	±8.47			47.1	±9.37			-0.09	±6.32		
Naïve	*$CD62L^+CD45RA^+$*	Negative	44.9	±9.51	0.73	0.398	47.6	±9.77	1.14	0.292	+2.62	±2.65	1.21	0.279
		Positive	40.6	±14.9			42.1	±15.2			+1.42	±3.10		
Central Memory	*$CD62L^+CD45RA^-$*	Negative	43.2	±6.79	0.40	0.529	40.4	±6.69	0.43	0.515	-2.83	±2.56	0.00	0.990
		Positive	40.8	±11.3			38.0	±10.9			-2.84	±2.57		
Effector memory	*$CD62L^-CD45RA^-$*	Negative	10.7	±4.03	3.59	0.066	10.9	±4.07	4.62	0.038	+0.17	±0.72	1.95	0.170
		Positive	15.4	±7.47			16.7	±8.16			+1.25	±2.41		
TEMRA	*$CD62L^-CD45RA^+$*	Negative	1.03	±0.94	3.36	0.074	1.19	±1.24	2.77	0.104	+0.15	±0.46	0.00	0.971
		Positive	3.31	±3.87			3.30	±3.91			+0.17	±1.34		
Activated	*HLA^-DR^+*	Negative	7.26	±3.21	0.82	0.369	6.15	±3.61	0.68	0.415	-1.12	±1.91	0.00	0.997
		Positive	8.34	±3.54			7.22	±3.90			-1.12	±2.53		
Senescent T helper cells	*$CD28^-$*	Negative	0.16	±0.13	6.16	0.017	0.17	±0.16	12.25	0.001	+0.01	±0.07	0.05	0.823
		Positive	8.31	±10.8			7.84	±7.21			-0.47	±7.04		
Non-senescent T helper cells	*$CD28^+$*	Negative	99.9	±0.14	6.15	0.017	99.9	±0.17	12.31	0.001	+0.00	±0.06	0.05	0.831
		Positive	91.7	±10.8			92.2	±7.21			+0.46	±7.04		
Regulatory T cells	*$CD4^+CD25^+Foxp3^+$*	Negative	7.65	±1.85	1.29	0.262	7.69	±1.51	3.72	0.060	+0.04	±1.51	0.94	0.336
		Positive	6.95	±1.78			6.58	±1.71			-0.32	±0.92		

**_Cytotoxic T cells_**	**_$CD3^+CD8^+$_**	Negative	15.4	±7.53	8.45	0.006	14.5	±6.33	18.5	0.000	-0.83	±2.27	0.76	0.387
		Positive	23.2	±7.93			24.4	±9.57			+0.18	±3.59		
Naïve	*$CD62L^+CD45RA^+$*	Negative	37.3	±11.4	16.8	0.000	36.3	±12.4	18.5	0.000	-1.03	±3.69	0.34	0.563
		Positive	22.8	±9.07			21.9	7.99			-0.26	±3.61		
Central Memory	*$CD62L^+CD45RA-$*	Negative	22.4	±9.13	2.07	0.159	21.2	8.36	0.00	0.989	-1.25	±3.67	0.00	0.944
		Positive	17.5	±9.54			15.7	9.18			-1.32	±2.69		
Effector memory cells	*CD62L-CD45RA-*	Negative	17.7	±5.90	0.20	0.655	17.9	6.26	0.00	0.989	+0.19	±2.96	1.36	0.252
		Positive	19.3	±10.2			17.9	9.77			-0.97	±2.65		
TEMRA	*$CD62L-CD45RA^+$*	Negative	22.5	±11.8	11.5	0.002	24.6	10.81	13.0	0.001	+2.12	±4.81	0.05	0.817
		Positive	40.5	±15.2			44.5	16.31			+2.56	±5.31		
Activated	*$HLA-DR^+$*	Negative	11.4	±5.68	1.23	0.274	12.2	6.85	0.32	0.572	+0.85	±3.38	0.78	0.382
		Positive	15.5	±11.8			14.3	11.75			-0.78	±5.80		
Senescent cytotoxic T cells	*CD28-*	Negative	24.9	±15.2	20.6	0.000	23.4	13.48	21.2	0.000	-1.51	±4.18	0.64	0.427
		Positive	51.8	±17.8			53.3	20.21			+0.36	±7.34		
Non-senescent cytotoxic T cells	*$CD28^+$*	Negative	75.1	±15.2	20.6	0.000	76.7	13.47	21.2	0.000	-0.00	±0.07	0.03	0.876
		Positive	48.2	±17.8			46.8	20.21			+0.36	±7.27		

Mean difference between baseline and follow-up, comparing the CMV negative group (11 patients) with the positive group (39 patients).

Supplementary Table S8. Complementary blood test results

Test (mmol/L)	Baseline	Follow-up		
	Mean (SD)	Mean (SD)	Mean difference	P
Sodium	141 (±2.40)	141 (±1.8)	+0.16	0.547
Potassium	4.17 (±0.28)	4.19 (±0.33)	+0.01	0.705
Calcium	2.37 (±0.09)	2.40 (±0.08)	+0.03	0.006
Phosphate	1.00 (±0.13)	1.04 (±0.15)	+0.05	0.005
Creatinine	86.2 (±17.4)	90.3 (±20.8)	+4.08	0.011
Glomerular filtration rate	70 (±13.2)	67 (±14.9)	-2.40	0.019
Urea	5.93 (±1.38)	6.32 (±1.61)	+0.39	0.009
Albumin	38.5 (±3.0)	39.0 (±2.9)	+0.56	0.840
Bilirubin	10.4 (±3.6)	11.2 (±5.5)	+0.76	0.225
ALP	1.15 (±0.42)	1.24 (±0.41)	+0.09	0.000
ASAT	0.48 (±0.11)	0.49 (±0.37)	+0.02	0.732
ALAT	0.48 (±0.20)	0.46 (±0.18)	-0.02	0.446
GT	0.58 (±0.79)	0.64 (±0.86)	+0.06	0.503

Blood test results, with results complementary to Table 3. The table shows results at baseline and at follow-up, 12 months later. Paired samples T test was used for differences over the study year.

Supplementary Table S9. Correlations between treatment effects and changes in NK- and NKT-like cell populations

**Significantly altered cell populations**		**LDL**		**Systolic blood pressure**		***Diastolic blood pressure***	
NK cells		*correlation*	P	*correlation*	P	*correlation*	P
Immature	*CD3-CD56^{bright}*	0.024	0.866	-0.037	0.798	-0.135	0.350
Mature	*CD3-CD56^{dim}*	-0.026	0.860	0.039	0.789	0.134	0.352
- Differentiated	*CD3-CD56^{dim}CD57^{+}*	-0.149	0.301	0.051	0.726	0.178	0.215
- CD85j^{+} (inhibitory receptor)	*CD3-CD56^{dim}CD85j^{+}*	0.049	0.736	-0.049	0.734	0.025	0.863
- NKG2C^{+} (activating receptor)	*CD3-CD56^{dim}NKG2C^{+}NKG2A-*	0.097	0.512	-0.036	0.808	-0.112	0.447
- NKG2A^{+} (inhibitory receptor)	*CD3-CD56^{dim}NKG2C-NKG2A^{+}*	-0.204	0.165	0.186	0.205	0.078	0.599
NKT-like cells							
- Differentiated	*CD3^{+}CD56^{+}CD57^{+}*	0.020	0.893	-0.021	0.884	0.275	0.530
CD8^{+} NKT-like cells	*CD3^{+}CD56^{+}CD8^{+}*	-0.072	0.618	-0.150	0.297	-0.022	0.881
- Differentiated	*CD3^{+}CD56^{+}CD8^{+}CD57^{+}*	-0.089	0.537	-0.047	0.747	0.027	0.851
T helper cells							
Naïve[a]	*CD62L^{+}CD45RA^{+}*	-0.016	0.922	0.030	0.855	-0.139	0.386
Central Memory[a]	*CD62L^{+}CD45RA-*	0.058	0.719	-0.069	0.667	0.288	0.068
Effector Memory[a]	*CD62L-CD45RA-*	-0.080	0.619	-0.086	0.593	-0.117	0.465
Activated	*HLA-DR^{+}*	0.002	0.988	-0.019	0.896	-0.175	0.223

Cytotoxic T cells							
Central Memory	*CD62L⁺CD45RA-*	-0.012	0.945	0.038	0.817	0.249	0.126
TEMRA	*CD62L⁻CD45RA⁺*	0.063	0.702	0.063	0.702	-0.195	0.234

Results from bivariate Pearson correlation analysis in all 50 patients, accounting for the relationships between numerically significant treatment effects and the immune cell populations that significantly changed over the study year. At group level, there were no significant correlations between any of the changes in cardiovascular risk factors and the cell populations in which differences were observed.

Supplementary Table S10. Medical treatment and treatment adjustments over the study year

	Baseline	Follow-up		
	N (%)	N (%)	Difference	P
**Anti-thrombotic agents**				
Anticoagulants	6 (12.0)	7 (14.0)	+ 1	0.79
Anti-platelet inhibitors	44 (88.0)	43 (86.0)	- 1	1.00
**Lipid Lowering agents**				
Simvastatin	20 (40.0)	10 (20.0)	- 10	0.11
Atorvastatin	17 (34.0)	24 (48.0)	+ 7	0.36
Rosuvastatin	6 (12.0)	11 (22.0)	+ 5	0.26
Ezetimibe	2 (4.0)	8 (16.0)	+ 6	0.07
**Anti-hypertensive treatment**				
ACE-I	30 (60.0)	33 (66.0)	+ 3	0.77
Calcium antagonist	23 (46.0)	22 (44.0)	- 1	1.00
Diuretics	17 (34.0)	18 (36.0)	+ 1	0.88
Betablockers	18 (36.0)	24 (48.0)	+ 6	0.44

Medical treatment and how it was adjusted over the study year. Data from all 50 participants. N= Number of patients with continuous dosage of the indicated drug.

Supplementary Table S11. Impediments for the implementation of optimal cardiovascular preventive treatment

	N (%)
Pain in joints/back/muscles	10 (20.0)
Intolerance for statins or other drugs	6 (12.0)
Unwilling to stop smoking	6 (12.0)
No motivation for changes in exercise patterns	5 (10.0)
Sequele or disabilities from diseases that limits exercise	4 (8.0)
Unwilling to medicine adjustments	4 (8.0)
Claudicatio or angina	3 (6.0)
Symptoms that limit blood pressure management	2 (4.0)
Difficulties in treating LDL due to Familial Hypercholesterolemia	1 (2.0)
Renal dysfunction and liver dysfunction that inhibits medicine adjustments	1 (2.0)
No impediments for optimization	8 (16.0)

Supplementary Table S12. Numbers and proportions of major lymphocyte populations at baseline and follow-up

		**Baseline** Mean (SD)	**Follow-up** Mean (SD)	**Difference** Mean (SD)	**P**
Lymphocytes	*%*[a]	24.4 (± 5.61)	25.2 (± 6.84)	+0.73 (± 5.14)	0.323
	x*10⁹/L*	1.80 (± 0.46)	1.85 (± 0.47)	+0.02 (± 0.35)	0.627
T cells	*%*[b]	74.0 (± 8.77)	74.6 (± 8.46)	+0.74 (± 3.86)	0.181
	x*10⁶/L*	1339 (± 405)	1378 (± 398)	+26.2 (±258)	0.476
T-helper cells	*%*	50.2 (± 9.61)	50.3 (± 9.60)	+0.36 (± 4.11)	0.539
	x*10⁶/L*	906 (± 283)	916 (± 252)	+3.60 (± 22.7)	0.875
Cytotoxic T cells	*%*	22.9 (± 9.61)	23.6 (± 9.94)	+0.45 (± 2.45)	0.205
	x*10⁶/L*	423 (± 226)	452 (± 260)	+21.3 (± 117)	0.204
B-cells	*%*	9.72 (± 3.88)	9.61 (± 4.35)	-0.164 (1.81)	0.525
	x*10⁶/L*	173 (± 79.8)	174 (± 88.0)	-1.72 (±34.8)	0.728
NK-cells	*%*	15.9 (± 7.73)	15.2 (± 7.74)	-0.74 (± 4.18)	0.219
	x*10⁶/L*	284 (± 151)	280 (± 176)	-6.82 (± 109)	0.659
$CD4^+/CD8^+$	*Ratio*	2.85 (± 2.31)	2.77 (±2.25)	-0.02 (± 0.43)	0.721

[a]Proportion of lymphocytes is expressed as % of leukocytes

[b]Proportions of major lymphocyte populations are expressed as % of lymphocytes

T cells	$CD3^+$
T-helper cells	$CD3^+4^+$
Cytotoxic T cells	$CD3^+8^+$
B-cells	$CD3^-19^+$
NK-cells	$CD3^-16^+56^+$

Supplementary Table S13. Natural Killer cells

**Population**			**Baseline**	**Follow-up**	**Difference**	**P**
Total NK-cells	*% of Ly*	*$CD3^-CD56^+$*	14.2 (± 7.1)	14.4 (± 7.2)	+0.1 (± 3.3)	0.810
Immature	*% of NK*	*$CD3^-CD56^{bright}$*	4.6 (± 3.0)	4.8 (± 3.0)	+0.2 (± 1.3)	0.385
Mature	*% of NK*	*$CD3^-CD56^{dim}$*	95.4 (± 3.0)	95.2 (± 3.0)	-0.2 (±1.3)	0.395
- Differentiated	*% of Mature NK*	*$CD3^-CD56^{dim}CD57^+$*	48.9 (± 18.5)	44.8 (± 18.2)	-4.4 (± 4.4)	0.000
- $NKG2C^+$ (activating receptor)	*% of Mature NK*	*$CD3^-CD56^{dim}NKG2C^+NKG2A^-$*	5.7 (± 8.4)	3.4 (± 7.5)	-2.0 (± 3.5)	0.000
- $NKG2A^+$ (inhibitory receptor)	*% of Mature NK*	*$CD3^-CD56^{dim}NKG2C^-NKG2A^+$*	39.4 (± 13.1)	40.7 (± 13.9)	+1.0 (± 3.1)	0.032
- $CD85j^+$ (inhibitory receptor)	*% of Mature NK*	*$CD3^-CD56^{dim}CD85j^+$*	42.0 (± 14.1)	41.7 (± 14.4)	-0.4 (± 3.1)	0.420
NKT-like cells	*% of Ly*	*$CD3^+CD56^+$*	9.1 (± 4.8)	10.5 (± 5.5)	+1.3 (± 2.4)	0.000
- Differentiated	*% of NKT*	*$CD3^+CD56^+CD57^+$*	54.9 (± 22.2)	47.3 (± 22.3)	-7.6 (± 10.0)	0.000
$CD8^+$ NKT-like cells	*% of NKT*	*$CD3^+CD56^+CD8^+$*	73.7 (± 15.6)	67.0 (± 17.5)	-6.7 (± 9.8)	0.000
- Differentiated	*% of NKT $CD8^+$*	*$CD3^+CD56^+CD8^+CD57^+$*	59.8 (± 22.1)	53.7 (± 22.4)	-6.1 (± 8.0)	0.000

NK cell and NKT-like cell response in all 50 patients comparing baseline and follow-up. All subgroups with significant changes are also presented in Figure 1A and 1B.

Supplementary Table S14. Proteins with a mean change (P<0.1, non-adjusted P-values) over the 12 study months

Top10	Gene	Protein	Uniprot ID	Difference (NPX)	Std. Deviation	Two-Sided P	Function
1	TRAIL*	TNF-related apoptosis-inducing ligand	P50591	+0.103	0.210	0.001	TNF-related apoptosis-inducing ligand.
2	HSP27*	Heat shock 27 kDa protein	P04792	-0.103	0.236	0.003	Associated with atherosclerotic process, e.g., marker of myocardial ischemia.
3	FS	Follistatin	P19883	+0.127	0.303	0.005	Autocrine glycoprotein involved in inflammatory response following tissue injury or repair.
4	LOX 1	Lectin-like oxidized LDL receptor 1	P78380	+0.186	0.508	0.013	Mediates recognition, internalization, and degradation of oxidatively modified LDL by vascular endothelial cells.
5	4E BP1	Eukaryotic translation initiation factor 4E-binding protein 1	Q13541	+0.563	1.546	0.015	Regulator of protein translation.
6	STK4	Serine/threonine-protein kinase 4	Q13043	-0.219	0.628	0.017	Stress-activated, pro-apoptotic kinase.
7	SRC	Proto-oncogene tyrosine-protein kinase Src	P12931	-0.151	0.441	0.019	Activated following engagement of different classes of cellular receptors including immune response receptors, integrins and other adhesion receptors, receptor protein tyrosine kinases, G protein-coupled receptors as well as cytokine receptors.
8	DCN	Decorin	P07585	-0.071	0.219	0.026	Regulation of TGF-beta activity. May function as an angiogenesis inhibitor.
9	GLO1	Lactoylglutathione lyase	Q04760	+0.374	1.164	0.028	Involved in regulation of TNF-induced transcriptional activity of NF-κB.
10	GDF 2	Growth/differentiation factor 2	Q9UK05	+0.122	0.383	0.028	Inhibitor of angiogenesis. Belongs to the transforming growth factor beta superfamily.
11	BOC	Brother of CDO	Q9BWV1	+0.073	0.228	0.029	Component of a cell-surface receptor complex that mediates cell-cell interactions between muscle precursor cells.
12	TRANCE*	Tumour necrosis factor ligand superfamily member 11	O14788	+0.136	0.427	0.032	Augments the ability of dendritic cells to stimulate naive T cell proliferation. May be an important regulator of interactions between T cells and dendritic cells and may play a role in the regulation of the T cell-dependent immune response.
13	TGM2*	Protein-glutamine gamma-glutamyltransferase 2	P21980	+0.254	0.835	0.036	Catalyses the cross-linking of proteins, such as WDR54, and the conjugation of polyamines to proteins.

14	TNFSF14	Tumour necrosis factor ligand superfamily member 14	O43557	+0.133	0.433	0.039	Delivers costimulatory signals to T cells, leading to T cell proliferation and IFNG production.
15	CCL19*	C-C motif chemokine 19	Q99731	+0.140	0.464	0.042	Involved in inflammatory and immunological responses. May play a key role in trafficking of T cells in thymus, and T cell and B-cell migration to secondary lymphoid organs.
16	THPO	Thrombopoietin	P40225	+0.080	0.270	0.042	Cytokine affecting the proliferation and maturation of megakaryocytes.
17	IL4RA	Interleukin-4 receptor subunit alpha	P24394	-0.067	0.236	0.051	Receptor for both interleukin 4 and interleukin 13. The IL4 response engages in promoting Th2 differentiation.
18	IL18R1	Interleukin-18 receptor 1	Q13478	+0.070	0.242	0.051	Involved in IL18-mediated IFNG synthesis from Th1 cells.
19	OSM	Oncostatin-M	P13725	+0.248	0.887	0.059	Growth regulator. Regulates cytokine production, including IL-6, G-CSF and GM-CSF from endothelial cells.
20	IL16	Pro-interleukin-16	Q14005	+0.083	0.306	0.060	Stimulates a migratory response in $CD4^+$ lymphocytes, monocytes, and eosinophils. Primes $CD4^+$ T cells for IL-2 and IL-15 responsiveness. Induces T-lymphocyte expression of interleukin 2 receptor. Ligand for CD4.
21	FGF 21	Fibroblast growth factor 21	Q9NSA1	-0.247	0.926	0.066	Stimulates glucose uptake in differentiated adipocytes.
22	FGF 23	Fibroblast growth factor 23	Q9GZV9	-0.104	0.391	0.066	Regulator of phosphate homeostasis. Regulator of vitamin-D metabolism.
23	TRAIL R2	Tumour necrosis factor receptor superfamily member 10B	O14763	-0.052	0.213	0.093	Receptor for TRAIL. Promotes the activation of NF-κB. Essential for ER stress-induced apoptosis.
24	ST1A1	Sulfotransferase 1A1	P50225	-0.148	0.603	0.096	Functions as a sulfonate donor to catalyse the sulphate conjugation of catecholamines, phenolic drugs and neurotransmitters.
25	CD4	T cell surface glycoprotein CD4	P01730	-0.054	0.229	0.099	Plays an essential role in the immune response and serves multiple functions in responses against both external and internal offenses. In T cells, functions primarily as a coreceptor for MHC class II molecule: peptide complex.

NPX, Normalized Protein Expression (log 2). Protein 1-16 show significant changes over the study year and protein 17-25 changes with P-value <0.1. Upregulation versus downregulation is shown in the column "difference", where NPX results from baseline are compared to NPX from follow-up.
* = Proteins associated with the NF-κB signalling pathway.

Supplementary Figure S1.

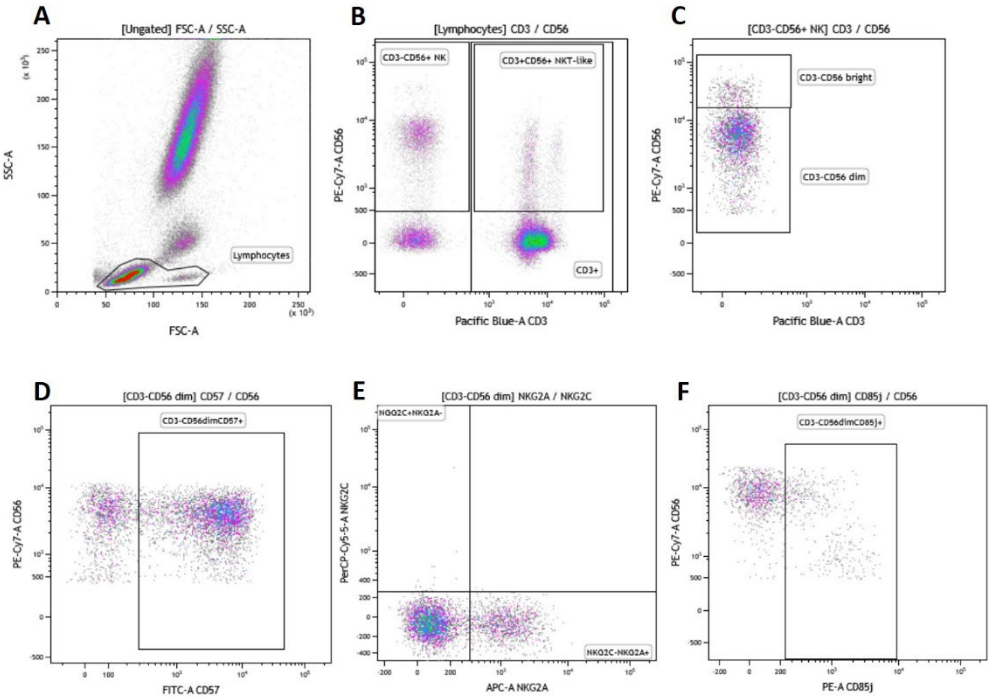

Gating strategy for natural killer (NK) cells, illustrated by representative dot plots. Lymphocytes were identified using forward scatter (FSC) and side scatter (SSC)(A). CD3 was plotted against CD56 to define NK-cells ($CD3^{-}CD56^{+}$) among lymphocytes (B). Mature ($CD56^{dim}$) and immature ($CD56^{bright}$) cells were determined within the NK-cell population (C). NK-cells were assessed for CD57 expression (D) and further classified by their expression of NKG2A/NKG2C (E) and CD85j (F).

Supplementary Figure S2.

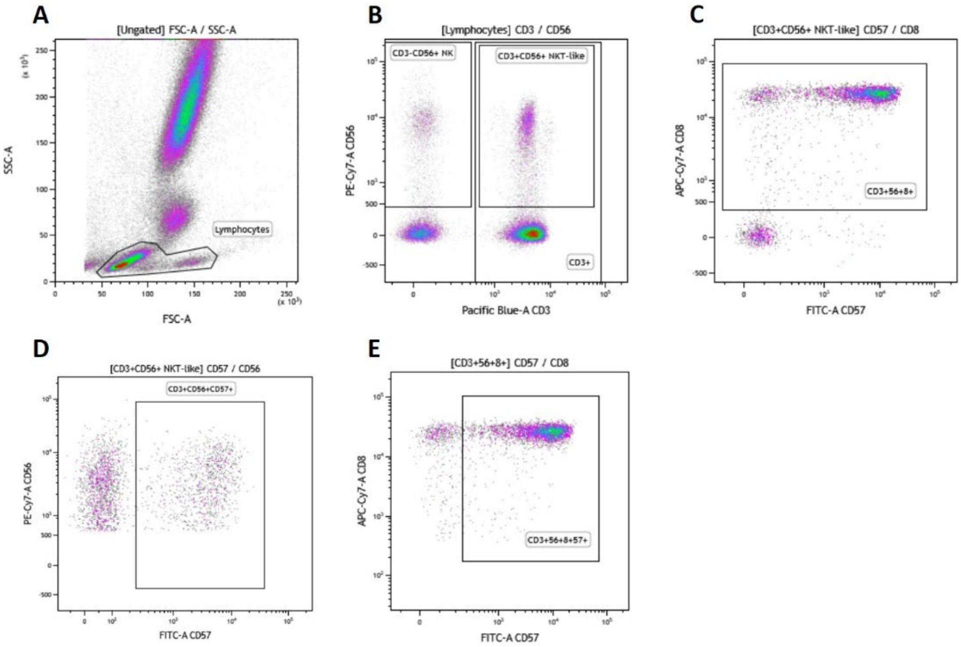

Gating strategy for natural killer T (NKT)-like cells, illustrated by representative dot plots. Lymphocytes were identified using forward scatter (FSC) and side scatter (SSC) (A). CD3 was plotted against CD56 to define NKT-like cells ($CD3^+CD56^+$) among lymphocytes (B) and expression of CD57 was assessed in the NKT-like population (C). $CD8^+$ NKT-like cells ($CD3^+CD56^+CD8^+$) were identified among total NKT-like cells (D) and CD57 was assessed in the $CD8^+$ NKT-like population as well (E).

Supplementary Figure S3.

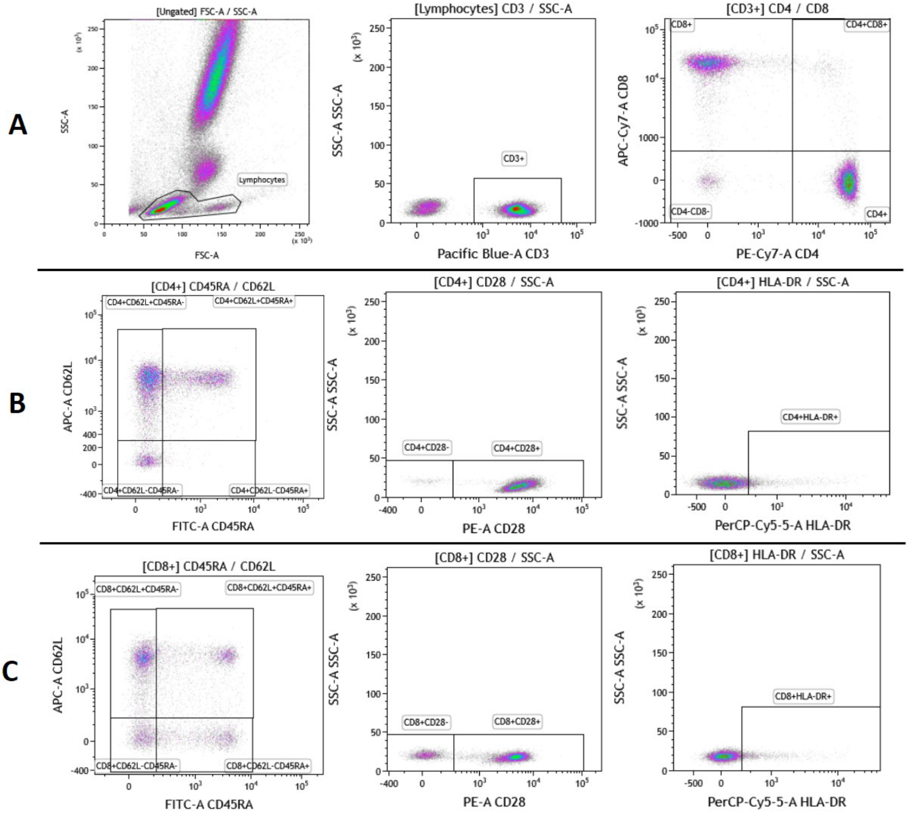

Gating strategy for T cell subsets, illustrated by representative dot plots. Panel A) Lymphocytes were identified using forward scatter (FSC) and side scatter (SSC). Among lymphocytes, T-cells were identified by their expression of CD3 and further classified into CD4$^+$ T helper cells and CD8$^+$ cytotoxic T cells.

Panel B) CD4$^+$ T cells were divided by their differentiation stage; naïve (CD45RA$^+$CD62L$^+$), central memory (T_{CM}, CD45RA$^-$CD62L$^+$), effector memory (T_{EM}, CD45RA$^-$CD62L$^-$) and terminally differentiated effector memory (T_{EMRA}, CD45$^+$CD62L$^-$), and the expression of CD28 and HLA-DR was determined in the total CD4$^+$ population. Panel C) CD8$^+$ T cells were analyzed in the same way as their CD4$^+$ counterpart, with all gate coordinates identical, with regard to naïve, T_{CM}, T_{EM} and T_{EMRA} populations as well as for CD28 and HLA-DR expression.

Supplementary Figure S4.

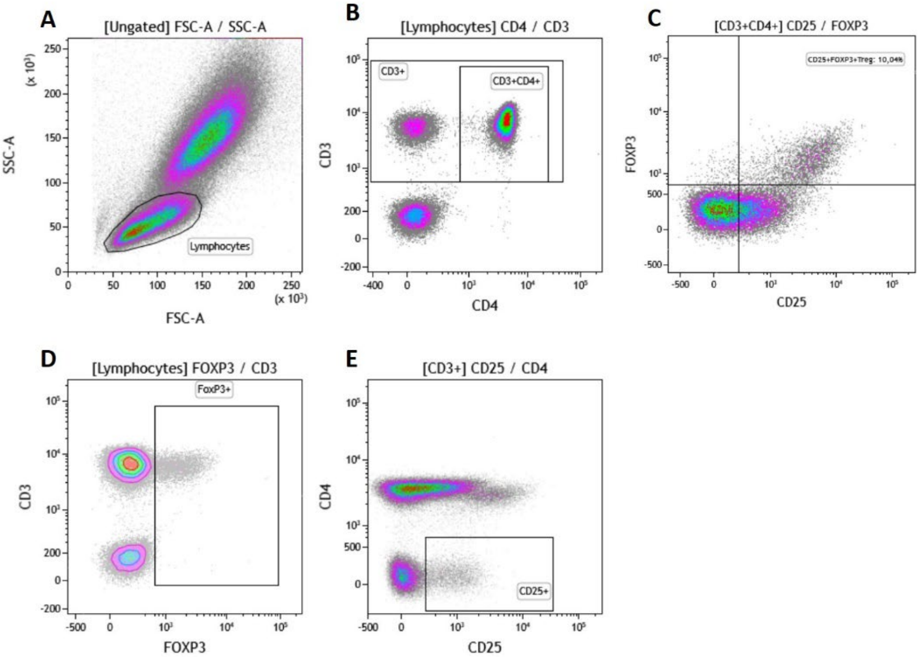

Gating strategy for regulatory T cells, illustrated by representative dot plots. Lymphocytes were identified using forward scatter (FSC) and side scatter (SSC) (A). Among lymphocytes, T helper cells were identified by their expression of CD3 and CD4 (B). The proportion of regulatory T cells (Treg, CD25$^+$FOXP3$^+$) was assessed within the CD4$^+$ population (C). A FOXP3$^-$ population among CD3$^-$ lymphocytes (D) was used to set the Treg FOXP3-gate (C), whereas the CD25$^-$ population among CD4- (i.e. CD8$^+$) T cells (E) was used to set the Treg CD25-gate (C).

Supplementary Figure S5.

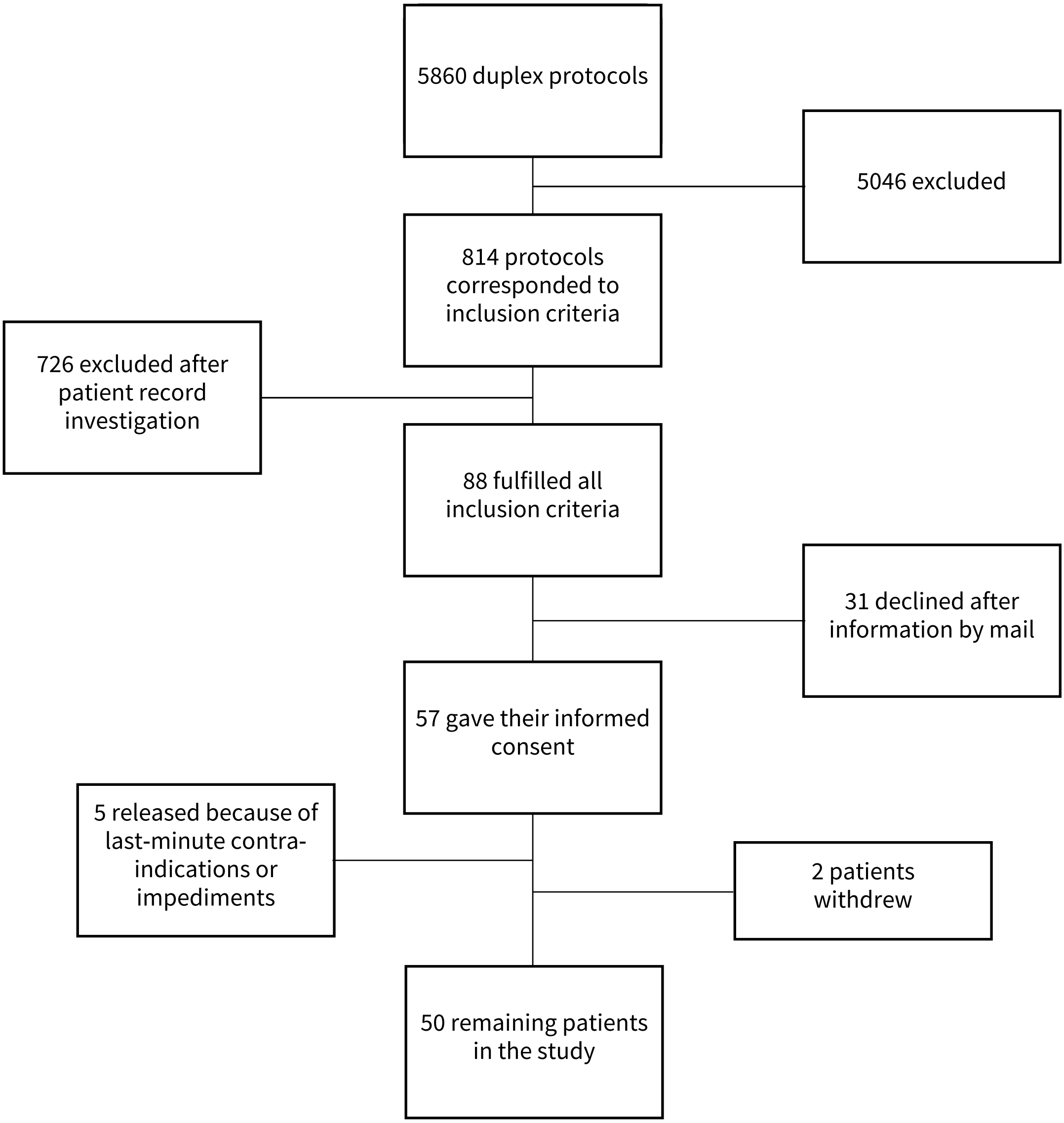

Inclusion flow chart showing inclusion process. In total, 5860 duplex protocols were checked for eligibility. After assessment of exclusion criteria and patient withdrawals, 50 patients remained in the study.

Supplementary Figure S6

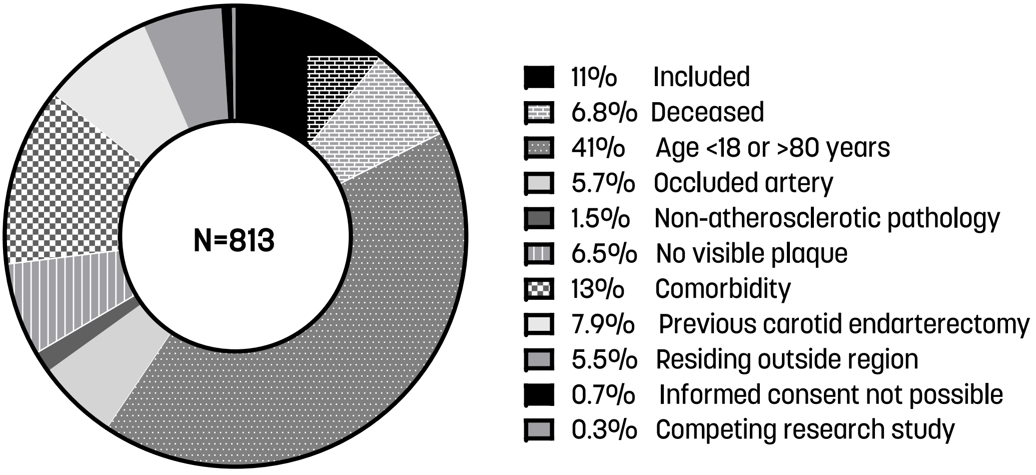

Reasons for exclusion of 726 study participants. Most common was age >80 years. In 6.5% the velocity in the carotid artery was increased on carotid ultrasound, but atherosclerotic changes were not observed. Comorbidities that inhibited study participation were present in 13%, and examples of such were kidney failure that prohibited medical optimisation and inflammatory diseases that were expected to influence study outcomes. Finally, 88 participants remained eligible for inclusion, whereof 50 remained in the study for both baseline and follow-up visits.
